# Impacts of aerosol direct effects on tropospheric ozone through changes in atmospheric dynamics and photolysis rates

**DOI:** 10.5194/acp-17-9869-2017

**Published:** 2017

**Authors:** Jia Xing, Jiandong Wang, Rohit Mathur, Shuxiao Wang, Golam Sarwar, Jonathan Pleim, Christian Hogrefe, Yuqiang Zhang, Jingkun Jiang, David C. Wong, Jiming Hao

**Affiliations:** 1 State Key Joint Laboratory of Environmental Simulation and Pollution Control, School of Environment, Tsinghua University, Beijing 100084, China; 2 The U.S. Environmental Protection Agency, Research Triangle Park, NC 27711, USA

## Abstract

Aerosol direct effects (ADEs), i.e., scattering and absorption of incoming solar radiation, reduce radiation reaching the ground and the resultant photolysis attenuation can decrease ozone (O_3_) formation in polluted areas. One the other hand, evidence also suggests that ADE-associated cooling suppresses atmospheric ventilation, thereby enhancing surface-level O_3_. Assessment of ADE impacts is thus important for understanding emission reduction strategies that seek co-benefits associated with reductions in both particuate matter and O_3_ levels. This study quantifies the impacts of ADEs on tropospheric ozone by using a two-way online coupled meteorology and atmospheric chemistry model, WRF- CMAQ, using a process analysis methodology. Two mani-festations of ADE impacts on O3 including changes in atmospheric dynamics (ᐃDynamics) and changes in photolysis rates (∆Photolysis) were assessed separately through multiple scenario simulations for January and July of 2013 over China. Results suggest that ADEs reduced surface daily maxima 1 h O_3_ (DM1O_3_) in China by up to 39μgm^−3^ through the combination of ∆Dynamics and ∆Photolysis in January but enhanced surface DM1O_3_ by up to 4μgm^−3^ in July. Increased O_3_ in July is largely attributed to ∆Dynamics, which causes a weaker O_3_ sink of dry deposition and a stronger O_3_ source of photochemistry due to the stabilization of the at-mosphere. Meanwhile, surface OH is also enhanced at noon in July, though its daytime average values are reduced in January. An increased OH chain length and a shift towards more volatile organic compound (VOC)-limited conditions are found due to ADEs in both January and July. This study suggests that reducing ADEs may have the potential risk of increasing O_3_ in winter, but it will benefit the reduction in maxima O_3_ in summer.

## 1 Introduction

Photochemistry in the atmosphere is a well-known source of tropospheric ozone (O_3_) (e.g., Haagen-Smit and Fox, 1954) and is determined by ambient levels of O_3_ precursors (i.e., NO_*x*_ and VOC) and photolysis rates, which are largely influenced by meteorological factors such as solar irradiance and temperature. It is well known that aerosols influence radiation through light scattering and absorption, thereby modulating atmospheric radiation and temperature. These aerosol direct effects (ADEs) can then impact thermal and photo-chemical reactions leading to the formation of O_3_ (Dickerson et al., 1997). Recent studies suggest that the aerosol-induced reduction in solar irradiance leads to lower photolysis rates and less O_3_ (e.g., Benas et al., 2013), and therefore extensive aerosol reductions, particularly in developing regions such as in East Asia, may pose a potential risk by enhancing O_3_ levels (Bian et al., 2007; Anger et al., 2016; Wang et al., 2016). For example, Wang et al. (2016) found that because of ADEs, the surface 1 h maximum ozone (noted as DM1O_3_) was reduced by up to 12 % in eastern China during the EAST-AIRE campaign, suggesting that the benefits of PM_2.5_ reductions may be partially offset by increases in ozone associated with reducing ADEs.

Ambient O_3_ levels are influenced by several sources and sinks. The modulation of photolysis rates by ADEs is only one manifestation of ADEs impacts on O_3_. In addition, ADEs modulate the temperature (e.g., Hansen et al., 1997; Mitchell et al., 1995), atmospheric ventilation (e.g., Jacobson et al., 2007; Mathur et al., 2010), cloud and rainfall (e.g., Albrecht, 1989; Liou and Ou, 1989; Twomey, 1977), which also influence the O_3_ concentrations. Therefore, ADEs can impact air quality through multiple pathways and process chains (Jacobson, 2002,^2010^; Jacobson et al., 2007; Wang et al., 2014; Xing et al., 2015a; Ding et al., 2016). For example, Xing et al. (2015a) suggested that the O_3_ response to ADEs is largely contributed by the increased precursor concentrations which enhance the photochemical reaction, presenting an overall positive response of O_3_ to ADEs by up to 2–3 % in eastern China. The assessment of a separate contribution from individual processes is necessary for fully understanding how ADEs impact O_3_.

In China, atmospheric haze is currently one of the most serious environmental issues of concern. Over the next decade, the national government plans to implement stringent control actions aimed at lowering the PM_2.5_ concentrations (Wang et al., 2017). Ideas on whether such extensive aerosol controls will enhance O_3_ and oxidation capacity needs to be carefully assessed and quantified. Many studies suggest that aerosols may have substantial impacts on ozone through heterogeneous reactions including hydrolysis of N_2_O_5_, irreversible absorption of NO_2_ and NO_3_, as well as the uptake of HO_2_ (Tang et al., 2004; Tie et al., 2005; Liao and Seinfeld, 2005; Pozzoli et al., 2008; Li et al., 2011; Xu et al., 2012; Lou et al., 2014). While our model contains comprehensive treatment of the heterogeneous hydrolysis of N_2_O_5_ (Davis et al., 2008; Sarwar et al., 2012, 2014), we have not quantified its impacts on ozone in this study. However, ADE impacts on ozone have not been well evaluated previously. Accurate assessment of the multiple ADE impacts is a prerequisite for accurate policy decision. The process analysis (PA) methodology is an advanced probing tool that enables quantitative assessment of integrated rates of key processes and reactions simulated in the atmospheric model (Jang et al., 1995; Zhang et al., 2009; Xu et al., 2008; Liu et al., 2010; Xing et al., 2011). In this study, we apply the PA methodology in the two-way coupled meteorology and atmospheric chemistry model, i.e., the Weather Research and Forecasting (WRF) model coupled with the Community Multiscale Air Quality (CMAQ) model developed by U.S. Environmental Protection Agency (Pleim et al., 2008; Mathur et al., 2010, 2014; Wong et al., 2012; Yu et al., 2014;; Xing et al., 2015b) to examine the process chain interactions arising from ADEs and quantify their impacts on O_3_ concentration.

The paper is organized as following. A brief description of the model configuration, scenario design and PA method is presented in Sect. 2. The O_3_ response to ADEs is discussed in Sect. 3.1. PA analyses are discussed in Sect. 3.2–3.3. The summary and conclusion is provided in Sect. 4.

## 2 Method

### 2.1 Modeling system

The two-way coupled WRF-CMAQ model has been detailed and fully evaluated in our previous papers (Wang et al., 2014; Xing et al., 2015a, b). The meteorological inputs for WRF simulations were derived from the NCEP FNL (Final) Operational Global Analysis data which has 1° spatial and 6 h temporal resolution. NCEP Automated Data Processing (ADP) Operational Global Surface Observations were used for surface reanalysis and four-dimensional data assimilation. We have tested and chosen the proper strength of nudging coefficients; i.e., 0.00005 s^−1^ is used for nudging both *u*/*v*-wind and potential temperature and 0.00001 s^−1^ is used for nudging the water vapor mixing ratio to improve model performance without dampening the effects of radiative feedbacks (Hogrefe et al., 2015; Xing et al., 2015b). In the model version used here, concentrations of gaseous species and primary and secondary aerosols are simulated by using Carbon Bond 05 gas-phase chemistry (Sarwar et al., 2008) and the sixth-generation CMAQ modal aerosol model (AERO6) (Appel et al., 2013). The aerosol optical properties were estimated by the coated-sphere module (i.e., BHCOAT; Bohren and Huffman, 1983) based on simulated aerosol composition and size distribution (Gan et al., 2015). In the coupled model, the estimated aerosol optical properties are fed to the RRTMG radiation module in WRF, thus updating the simulated atmospheric dynamics which then impact the simulated temperature, photolysis rate, transport, dispersion, deposition, cloud mixing and removal of pollutants. Due to large uncertainties associated with the representation of aerosol impacts on cloud droplet number and optical thickness, the indirect radiative effects of aerosols are not included in the current calculation.

**Figure 1.**
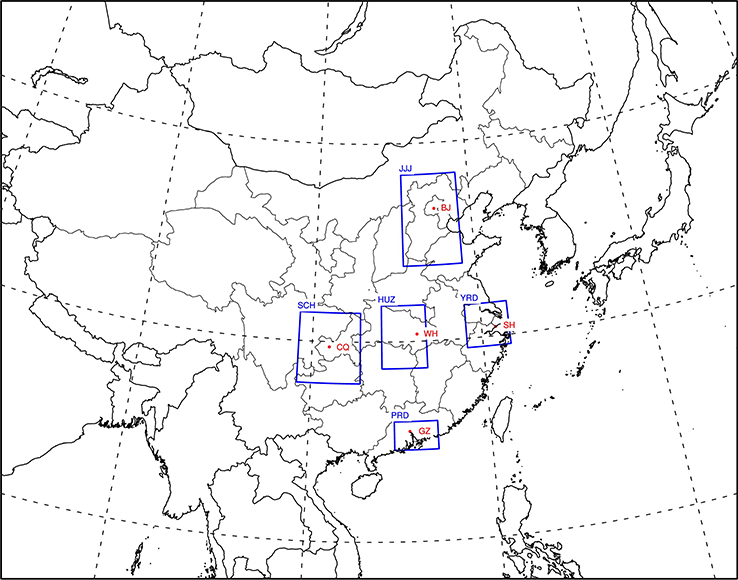
Simulation domain and locations of five selected regions in China. Note: JJJ: Jing-Jin-Ji area; YRD: Yangzi River Delta area; PRD: Pearl River Delta area; SCH: Sichuan Basin area; HUZ: Hubei-Hunan area.

The gridded emission inventory and initial and boundary conditions are consistent with our previous studies (Zhao et al., 2013a, b; Wang et al., 2014), while the simulated domain is extended slightly to cover all of China, as shown in Fig. 1. A better model performance in the simulation of dynamic fields including total solar radiation, planetary boundary layer (PBL) height data as well as PM_2.5_ concentrations was suggested after the inclusion of ADEs (Wang et al., 2014). In this study, the model performance in the simulation of O_3_ will be evaluated through the comparison with observations from 74 cities across China from the China National Urban Air Quality Real-time Publishing Platform (http://113.108.142.147:20035/emcpublish/). The simulation period is selected as 1 to 31 January and 1 to 31 July in 2013 to represent winter and summer conditions, respectively. Five regions are selected for analysis, including the Jing-Jin-Ji area (denoted JJJ), the Yangzi River Delta (denoted YRD), the Pearl River Delta (denoted PRD), the Sichuan Basin (denoted SCH) and the Hubei-Hunan area (denoted HUZ), as shown in Fig. 1.

### 2.2 Simulation design

Table 1 summarizes the scenario design in this study. In the baseline simulation (denoted SimBL), no aerosol feedbacks either on photolysis rates or radiation were taken into account. In simulation SimNF, only aerosol feedbacks on photolysis rates were considered by embedding an inline photolysis calculation in the model which accounted for the modulation of photolysis due to ADEs. Finally, in simulation SimSF aerosol feedbacks were considered on both photolysis rates and radiation calculations. Differences between the simulations of SimNF and SimBL are considered as ADE impacts on O_3_ through photolysis (denoted ∆Photolysis). Similarly, differences between the simulations of SimSF and SimNF are considered as the ADE impacts on O_3_ through dynamics (denoted ∆Dynamics), and differences between the simulations of SimSF and SimBL represent the combined ADE impacts on O_3_ due to both photolysis and dynamics (denoted ∆Total).

### 2.3 Process analysis

In this study the PA methodology is used in the WRF-CMAQ model to analyze processes impacting simulated O_3_ level. The integrated process rates (IPRs) track hourly contributions to O_3_ from seven major modeled atmospheric processes that act as sinks or sources of O_3_. These processes are gas-phase chemistry (denoted CHEM), cloud processes (i.e., the net effect of aqueous-phase chemistry, below- and in-cloud scavenging, and wet deposition, together denoted CLDS, dry deposition (denoted DDEP), horizontal advection (denoted HADV), horizontal diffusion (denoted HDIF), vertical advection (denoted ZADV) and turbulent mixing (denoted VDIF). The difference in IPRs among SimBL, SimNF and SimSF represents the response of individual process to ADEs. To enable the consistent examination of changes in the process due the ADEs across all concentration ranges, we examine changes in the IPRs normalized by the O_3_ concentrations. The differences in these process rates (expressed in units h^−1^) between the SimBL, SimSF and SimNF then provide estimates of the changes in process rates resulting from ADEs and are shown in the column (b) of Figs. 4 and 6 and (b)-(d) of Fig. 5.

Integrated reaction rates (IRRs) are used to investigate the relative importance of various gas-phase reactions in O_3_ formation. Following the grouping approach of previous studies (Zhang et al., 2009; Liu et al., 2010; Xing et al., 2011), the chemical production of total odd oxygen (O_*x*_) and the chain length of hydroxyl radical (OH) are calculated. Additionally, the ratio of the chemical production rate of H_2_O_2_ to that of HNO_3_ (*P*H_2_O_2_/*P*HNO_3_) is an estimated indicator of NO_*x^−^*_ or VOC- limited conditions for O_3_ chemistry.

## 3 Results

### 3.1 O_3_ response to ADEs

**Figure 2.**
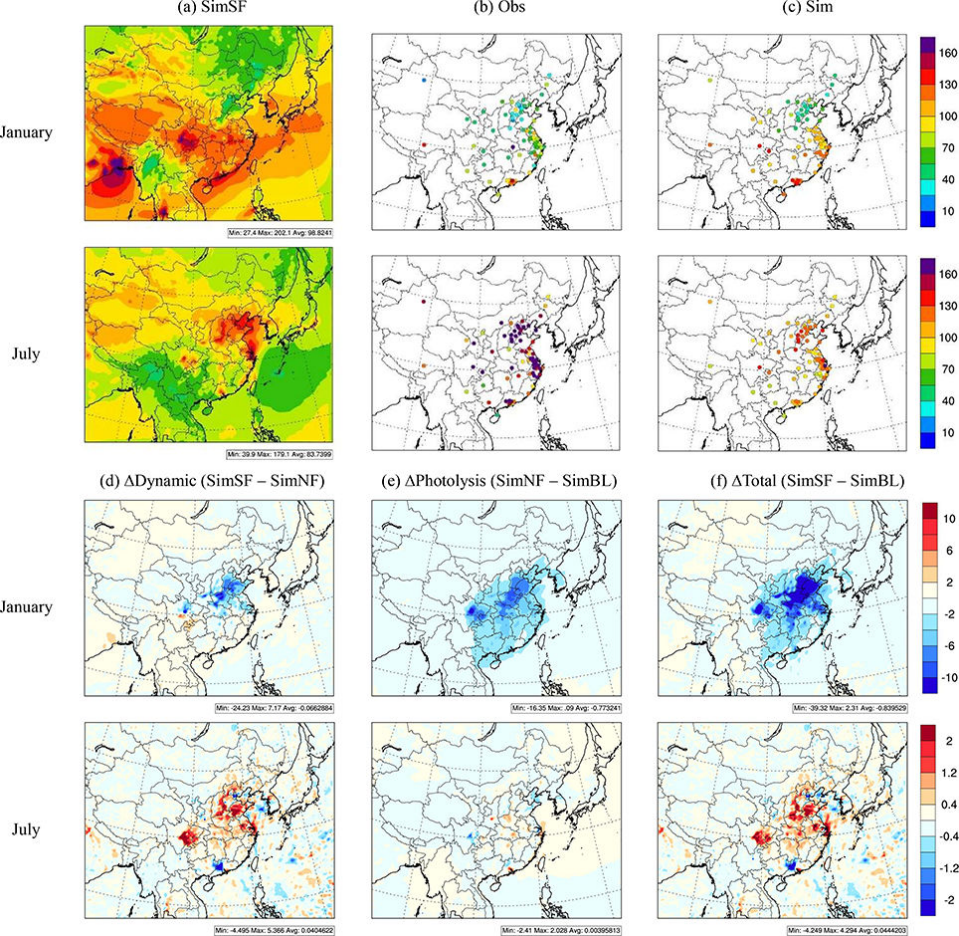
Observed and simulated O_3_ and its response to ADEs (monthly average of daily 1 h maxima, μg m ^−3^).

The simulated surface DMIO_3_ in SimBL, SimNF and SimSF is compared in Fig. 2a-c. In January, higher DM1O_3_ concentrations are seen in PRD, where solar radiation is stronger than in the north. The model generally captured the spatial pattern with highest DM1O_3_ in PRD over the simulated domain. Simulated DMIO_3_ in YRD, SCH and HUZ is higher than observations. Such overestimation might be associated with the relatively coarse spatial resolution in the model. NO titration effects in urban areas were not well represented in the model. In July, high DM1O_3_ areas are located towards the north, especially in the JJJ and YRD regions, which have relatively larger NO_*x*_ and VOC emission density and favorable meteorological conditions (e.g., less rain and moderate solar radiation).

In January, O_3_ production in north China is occurs in a VOC-limited regime (e.g., Liu et al., 2010); thus, increases in NO_*x*_ at the surface stemming from the stabilized atmosphere by ADEs (Jacobson et al., 2007; Mathur et al., 2010; Ding et al., 2013; Xing et al., 2015) inhibit O_3_ formation due to enhanced titration by NO. As seen in Fig. 2d, the ∆Dynamics reduced DM1O_3_ in eastern China by up to 24μgm^−3^ but slightly increased DM1O_3_ in parts of southern China by up to 7 μg m^−3^. The decrease in incoming solar radiation due to ADEs significantly reduces the photolysis rates in east China. As seen in Fig. 2e, the ∆Photolysis reduced DM1O_3_ domain-wide by up to 16μgm^−3^. The combined effect of both ∆Dynamics and ∆Photolysis results in an overall reduction in DM1O_3_ as evident across the JJJ and SCH regions with monthly-average reductions of up to 39 μg m^−3^.

In July, the O_3_ chemistry changes from a VOC-limited to an NO_*x*_ -limited regime across most of China. Therefore, an increase in NO_*x*_ concentration due to the stabilization of the atmosphere associated with the ADEs, facilitates O_3_ formation. The ∆Dynamics increased DM1O_3_ across most areas of China, particularly in JJJ, YRD and SCH by up to 5 μg m^−3^, with the exception of the PRD region where DM1O_3_ decreased. The APhotolysis results in contrasting impacts in July compared to January, as it increased DM1O_3_ in most polluted areas including JJJ, YRD, PRD, HUZ, although the solar radiances were reduced due to ∆Photolysis. This behavior is likely due to enhanced aerosol scattering associated with higher summertime $SO_{4}^{2-}$ levels (He and Carmichael, 1999; Jacobson, 1998). Similar results were found in Tie et al. (2005), who reported that surface-layer photolysis rates in eastern China were reduced less significantly in summer than in winter. The resultant enhancements in photolysis rates can then cause the noted higher concentrations. More importantly, the diurnal analysis (discussed in the next section) suggested that the reduced photolysis during the early morning in SimNF enhances the ambient precursor concentrations (due to less reaction in the early morning) at noon when O_3_ reaches the daily maximum. This increase in precursor concentrations then leads to enhanced O_3_ formation later in the day which compensates for or even outweighs the disbenefit from the reduced solar radiances. In summer, ∆Dynamics results in a much stronger influence on DM1O_3_ than ∆Photolysis, and the combined impact of ADEs increased O_3_ in most of regions in China by up to 4 μg m^−3^.

**Figure 3.**
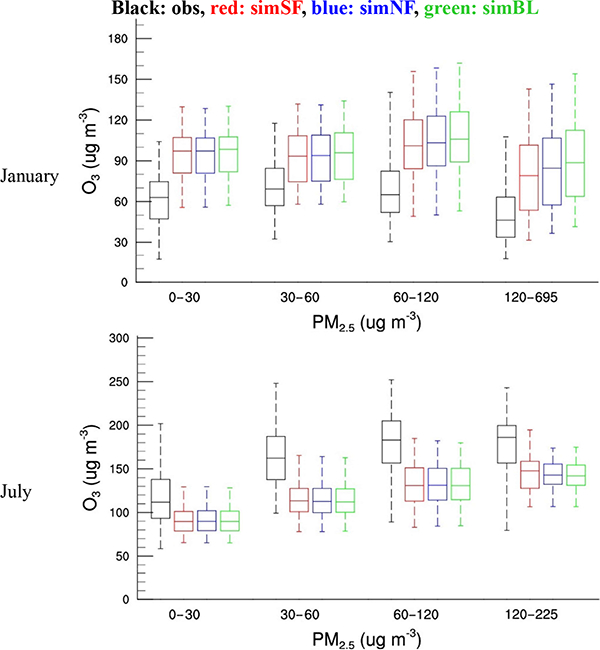
Observed and simulated surface O_3_ concentration against PM_2.5_ concentration (O_3_ is daily 1 h maximum of monitoring sites over China - unit: μg m^−3^; PM_2.5_ is the daily average of those site - unit: μgm^−3^).

The impact of the ADEs on O_3_ is further explored by examining the relationship between the observed and simulated O_3_ concentrations (DM1O_3_, daily values of the cities located in China) as a function of the observed PM_2.5_ concentrations (observed daily averaged values in those cities), as displayed in Fig. 3. The predicted ozone concentrations under both low and high PM_2.5_ levels are compared in Table 2. In regards to model performance for DM1O_3_ simulations, the model generally exhibits a slight high bias in January but a low bias in July across the five regions. The inclusion of ADEs moderately reduced O_3_ concentrations in January and slightly increased O_3_ in July, resulting in a reduction in bias and improved performance for DM1O_3_ simulation in both January and July for most of the regions. Comparing the O_3_ responses to ADEs (see ∆-ADE in Table 2) under low and high PM_2.5_ levels reveals that the O_3_ responses to ADEs are larger under high PM_2.5_ levels, indicating the positive correlations between O_3_ responses and PM_2.5_ levels.

Interestingly, from low to moderate PM_2.5_ levels (i.e., PM_2.5_ < 120 μgm ^−3^), higher O_3_ concentration occur with higher PM_2.5_ concentrations, which is evident in both observations and simulations, suggestive of common precursors (e.g., NO*_x_*), source sectors and/or transport pathways contributing to both O_3_ and PM_2.5_ in these regions. However, a negative correlation between O_3_ and PM_2.5_ is evident in winter when PM_2.5_ can reach high levels larger than 120 μg m^−3^, indicating the strong ADE impacts on O_3_ through both feed- backs to dynamics and photolysis which significantly reduced O_3_.

### 3.2 IPRs response to ADEs

**Figure 4.**
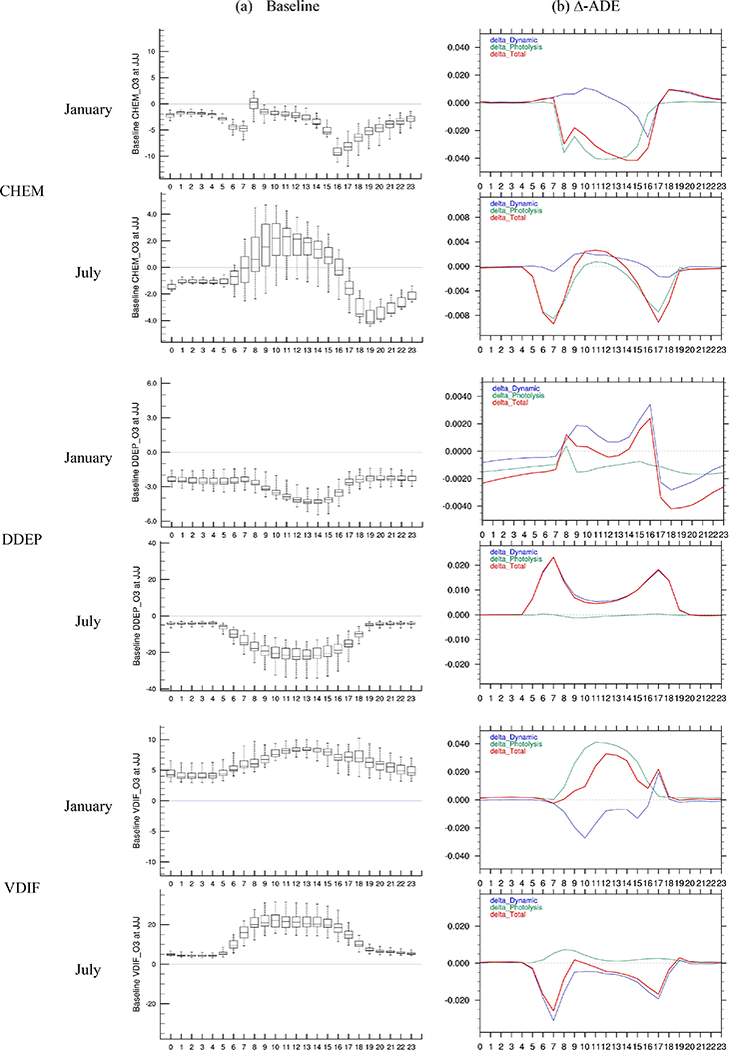
Diurnal variation in selected integrated process contributions to surface O_3_ concentration in JJJ. The calculation is based on the average of grid cells in JJJ; **(a)** baseline is the simulated O_3_ in SimBL (unit: ppbh^−1^); **(b)** ∆-ADE is the difference in normalized IPRs between simulations (unit: h^−1^). Delta_Dynamic is the difference between SimSF and SimNF; delta_Photolysis is the difference between SimNF and SimBF; delta_Total is the difference between SimSF and SimBF).

**Figure 5.**
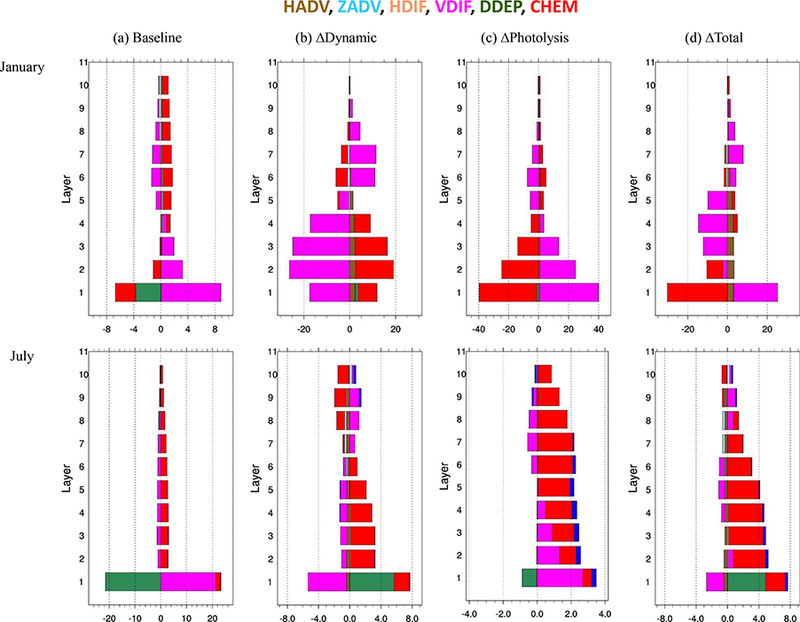
Vertical profile of integrated process contributions to surface O_3_ concentration at noon in JJJ. Full-layer heights above ground are 40, 96, 160, 241, 355, 503, 688, 884, 1100 and 1357m; **(a)** baseline is the simulated O_3_ in SimBL (unit: ppbh^−1^); **(b)** ∆Dynamic is the difference in normalized IPRs between SimSF and SimNF (unit: h^−1^); **(c)** ∆Photolysis is the difference in normalized IPRs between SimNF and SimBL (unit: h^−1^); **(d)** ∆Total is the difference in normalized IPRs between SimSF and SimBL (unit: h^−1^).

**Figure 6.**
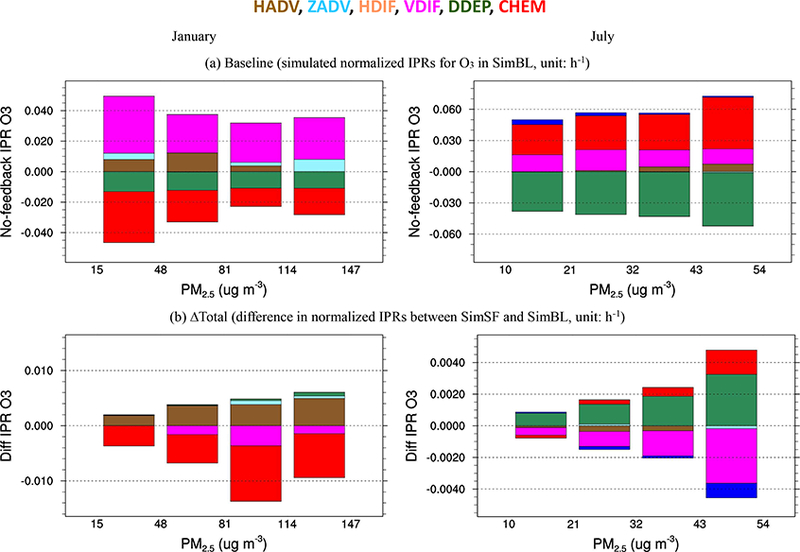
Integrated process contributions to daytime near-ground-level O_3_ under different PM_2.5_ levels in III (between the ground and 350ma.g.l.; model layers 1–5).

To further explore the ADE impacts on simulated O_3_, the integrated process contributions are further analyzed in three ways: (a) 24 h diurnal variations in process contributions to simulated surface O_3_ (Fig. 4); (b) vertical profiles from ground up to 1357 m a.g.l. (above ground level, in model layers 1–10) at noon (Fig. 5); and (c) correlations with nearground PM_2.5_ (average concentrations between the ground and 355 m a.g.l.; model layers 1–5) (Fig. 6). In the following, we limit our discussion to the analysis of model results for the JJJ region, which exhibited the strongest ADEs among the regions; similar results were found for the other four regions and can be found in the Supplement.

Diurnal variation in process contributions from chemistry (CHEM), dry deposition (DDEP) and vertical turbulent mixing (VDIF), which together contribute to more than 90 % of the O_3_ rate of change for the JJJ region, are illustrated in Supplement for JJJ and Figs. S2−S5 for the other four regions.

For surface-level O_3_, VDIF is the major source and DDEP is the major sink (Fig. S1). The stabilization of the atmosphere due to ∆Dynamics leads to lower dry deposition rates (due to lower dry deposition velocity from the enhanced aerodynamic resistance) and thus increases surface O_3_. The largest impact of ∆Dynamics on DDEP occurs during early morning and late afternoon, which is consistent with the response of the PBL height to ADEs noted in our previous analysis (Xing et al., 2015a).

As expected, CHEM is the second-largest sink for surface O_3_ during January but a source of surface O_3_ during the daytime in July. The ∆Dynamics increased the surface O_3_ around noon in both January and July for almost all regions (no impacts in PRD and YRD in January; see Figs. S2-S3), since increased stability due to ∆Dynamics concentrated more precursors locally, leading to enhanced O_3_ formation during the photochemically most active period of the day. The ∆Dynamics reduced the surface O_3_ around late afternoon in January in all regions. This is because the increased atmospheric stability during late afternoon and evening hours increased NO_*x*_ concentration, which titrated more O_3_. The ∆Photolysis reduced surface O_3_ in all regions in January. These reductions were more pronounced during the early morning hours when the photolysis rate are most sensitive to the radiation intensity. The ∆Photolysis resulted in comparatively larger reductions in surface O_3_ during the early morning and late afternoon hours in July but slightly increased surface O_3_ at noon for most of the regions. This increase in O_3_ can be hypothesized to result from the following sequence of events. Slower photochemical reaction in the morning in the ∆Photolysis case leads to higher levels of precursors, whose accumulation then enhances O_3_ formation at noon. This hypothesis is further confirmed by the changes in the diurnal variation in NO_2_, which suggest that higher NO to NO_2_ conversion during early morning results in enhanced daytime NO_2_ levels (see Fig. S6), consequently leading to higher noontime O_3_.

For O_3_ aloft (from 100 to 1600 m above ground), as seen in Fig. 5, CHEM is the major source of O_3_ at noon both in January and in July. The ∆Dynamics increased near-surface O_3_ (below 500 m; model layers 1–6) but reduced upper-level O_3_ (above 500 m; model layers 7–10) because increased stability of the atmosphere concentrated precursor emissions within a shallower layer resulting in higher O_3_ production. The ∆Photolysis case considerably reduced near-surface O_3_ at noon in January. In July, ∆Phololysis increased upper- level O_3_ at noon. Higher levels of precursors at noon might be the reason for such enhancement (see Fig. S6).

The daytime near-ground-averaged (between the ground and 350ma.g.l.; layers 1–5) IPR responses to ADEs are shown in Fig. 6 for JJJ and in Fig. S7 for other regions. The IPR and its responses are presented as a function of nearground-averaged PM_2.5_ concentrations. As shown in Fig. 6, as PM_2.5_ concentrations increase, the positive contribution of CHEM in July becomes larger, while the negative contribution of CHEM in January becomes smaller. The overall ADEs enhanced CHEM and thus increased O_3_ concentration in July, and such enhancement is generally larger for higher PM_2.5_ loading. In contrast, in January overall ADEs resulted in higher rates of O_3_ destruction due to chemistry (negative contribution of CHEM), and the magnitude of this sink increased as PM_2.5_ concentrations increase. The reduction of O_3_ steimning from the enhancements in the chemical sinks is the dominant impact of ADEs in January. The enhanced positive contribution of CHEM due to ∆Dynamics was partially compensated for by the reduction from ∆Phololysis (see Fig. S7), resulting in a slight increase in the positive CHEM contribution to O_3_ in July.

DDEP is the major sink of daytime O_3_ during both January and July. The increased stability due to ADEs reduced deposition velocity and thus increased O_3_. These effects become larger with increasing PM_2.5_ concentrations. Thus, weaker removal of O_3_ from DDEP associated with ADEs contributed to higher O_3_ in most regions during both January and July. An enhanced O_3_ source of CHEM and reduced O_3_ sink of DDEP is the dominant impact of ADEs in July.

### 3.3 IRR response to ADEs

**Figure 7.**
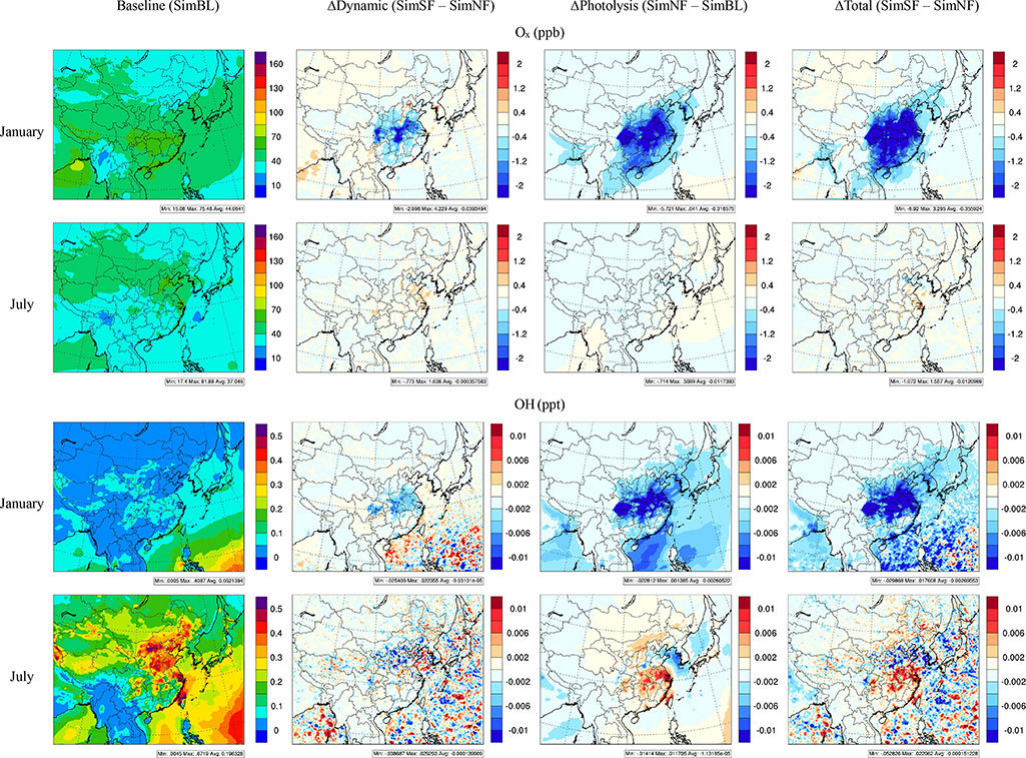
Impacts of ADEs on surface O_*x*_ and OH (monthly average of noon time 11:00–13:00 local time).

The simulated midday average (11:00–13:00 local time) surface O_*x*_ (defined as the sum of Ο, O_3_, NO_2_, NO_3_, N_2_O_5_, HNO_3_, peroxynitric acid, alkyl nitrates and peroxyacyl nitrates) and OH and their responses to ADEs is shown in Fig. 7. Both Of and OH are significantly reduced in the ∆Photolysis case in January throughout the modeling domain. Both Of and OH also show reductions in the middle portions of east China in the ∆ Dynamics case in January. Together, the combined ADE impacts result in reduced O_*x*_ and OH in January, with widespread reductions primarily due to ADEs on photolysis. In July, APhotolysis increased mid-day OH across most of China (Fig. 7), which is consistent with the increase in O_3_ at noon steimning from a higher level of precursor accumulation due to ∆Photolysis. The overall ADE impact on OH is controlled by ∆Photolysis and results in increased midday OH across most of China. For O_*x*_, how-ever, the impact of ∆ Dynamics outweighs the impact from ∆Photolysis, resulting in increase in O_*x*_ concentrations in east China including YRD, SCH and HUZ.

**Figure 8.**
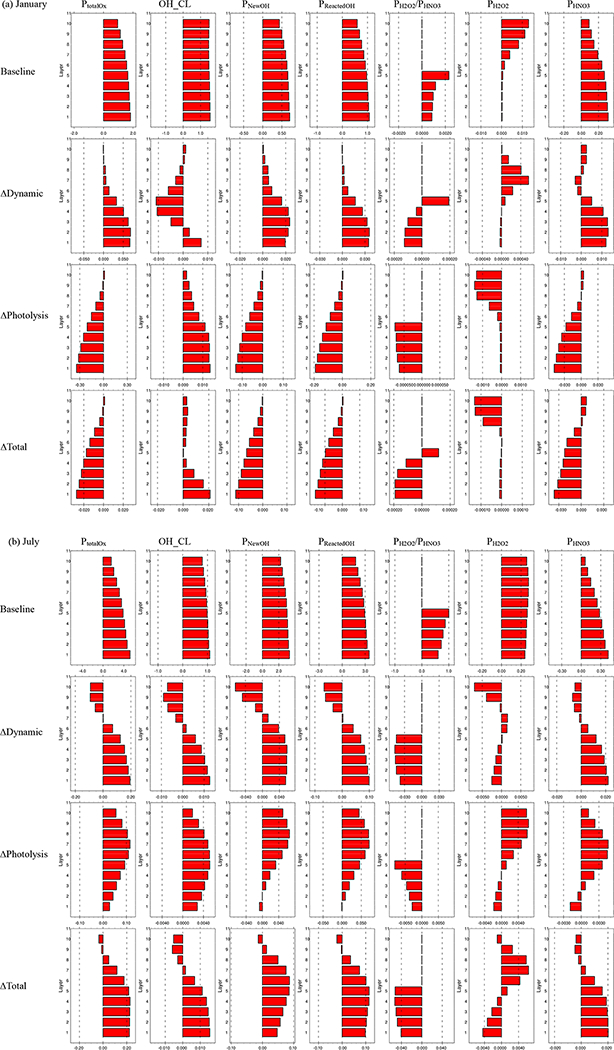
Vertical profile of integrated reaction rates in JJJ at noon. Full-layer heights above ground are 40, 96, 160, 241, 355, 503, 688, 884, 1100 and 1357 m; baseline is the simulation in SimBL; ∆Dynamic is the difference between SimSF and SimNF; ∆Photolysis is the difference between SimNF and SimBL; ∆Total is the difference between SimSF and SimBL; P_totalO_*x*__ is total O_*x*_ production rate (unit: ppbh^−1^); OH_CL is OH chain length; *P*_NewOH_ is the production rate of new OH (unit: ppbh^−1^); *P*_ReactedOH_ is the production rate of reacted OH (unit: ppbh^−1^); *P*_h2O2_ is the production rate of H_2_O_2_ (unit: ppbh^−1^); *P*_hno3_ is the production rate of HNO3 (unit: ppbh^−1^); the ratio of *P*_h2O2_/P_hno3_ is only shown for layers 1–5.

To further examine the response of O_*x*_ to ADEs, in Fig. 8 we examine vertical profiles of the integrated reaction rates at noon for the JJJ region. The stabilization of the atmosphere due to ∆ Dynamics concentrates precursors within a lower PBL, resulting in an increased total O_*x*_ production rate (*P*_totalO*x*_) mostly in near-ground model layers (below 500 m; model layers 1–6); in magnitude aloft (above 500 m; model layers 7–10), this change in *P*_totalO*x*_ is smaller in January and becomes decreasing in July. The reduction of *P*_totalO_*x*__ due to ∆Photolysis is greatest at the surface in January and declines with altitude and even becomes reversed at high layers (about 1300m; model layer 10) (Fig. 8a). The overall ADE impact in January is mainly dominated by ∆Photolysis, which largely outweighs the impact of ∆ Dynamics (Fig. 8a). How-ever, in July (Fig. 8b), ∆Photolysis enhanced *p*_totalO*x*_ across all layers. The -*P*_totalO_*x*__ shows small decreases at high altitudes but a significant increase in near-ground model layers (below 500 m; model layers 1–6) due to the combined ADEs in July.

The changes in vertical profiles of production rates of new OH (*P*_NewOH_) and reacted OH (*P*_ReactedOH_) are similar to those of *P*_totalO_*x*__, with the noted decreases in January dominated by ∆Photolysis. In contrast, the increases in July result from contributions from both ∆Photolysis and ∆ Dynamics.

An analysis of the chain length is important to understand the characteristics of chain reaction mechanisms. The OH chain length (denoted OH_CL) is determined by the ratio of *P*_ReactedOH_ to *P*_NewOH_· ∆ Dynamics concentrated more NO_*x*_ at the surface, thus leading to an increased OH_CL (i.e., more reacted OH than new OH) in the near-ground layers but a decreased OH_CL in the upper layers. In January, the ∆Photolysis reduced *P*_NewOH_ more than *P*_ReactedOH_ (probably because of more abundance of NO_*x*_ resulting from photolysis attenuation and consequently reduced photochemistry), thereby leading to an increased OH_CL. In July, ∆Photolysis enhanced both *P*_NewOH_ and *P*_ReactedOH_, particularly in the upper layers. The OH_CL is increased by ∆Photolysis because higher NO_*x*_ levels (see Fig. S6) cause more OH to be reacted. Thus the surface OH_CL at noon is increased in both January and July from combined ADEs of ∆Photolysis and ∆ Dynamics, indicating a stronger propagation efficiency of the chain.

The production rates of H_2_O_2_ (*P*_H_2_O_2__) and HNO_3_ (*P*_HNO_3__) and their responses to ADEs are also suimnarized in Fig. 8 (average for midday hours) for the JJJ region (similar illustrations for the other regions can be found in the supplemental Figs. S8-S11. Smaller ratios of *P*_H_2_O_2__/*P*_HNO_3__are noted in January compared to July, indicating a stronger VOC-limited regime in January for all regions. The ∆ Dynamics increases *P*_HNO_3__ but decreases *P*_H_2_O_2__ in both January and July because the enhanced NO_*x*_ at the surface in a more stable atmosphere likely shifts O_3_ chemistry towards NO_*x*_-rich conditions. The ∆Photolysis reduced both *P*_H_2_O_2__ and *P*_HNO_3__, but the ratio of *P*_H_2_O_2__/*P*HNO_3_ is decreased due to a larger reduction in *P*_H_2_O_2__ than *P*_HNO_3__. The combined impacts of ∆ Dynamics and ∆Photolysis result in a shift towards more VOC-limited conditions in the near-surface layers during both January and July.

## 4 Summary

The impacts of ADEs on tropospheric ozone were quantified by using the two-way coupled meteorology and at-mospheric chemistry WRF-CMAQ model using a process analysis methodology. Two manifestations of ADE impacts on O_3_ - changes in atmospheric dynamics (∆Dynamics) and changes in photolysis rates (∆Photolysis) - were systematically evaluated through simulations that isolated their impacts on modeled process rates over China for winter and summer conditions (represented by the months of January and July in 2013, respectively). Results suggest that the model performance for surface DM1O_3_ simulations improved after the inclusion of ADEs, which moderately reduced the high bias in January and low bias in July. In winter, the inclusion of ADE impacts resulted in an overall reduction in surface DM1O_3_ across China by up to 39 μg m^−3^. Changes both in photolysis and atmospheric dynamics due to ADEs contributed to the reductions in DM1O_3_ in winter. In contrast during July, the impact of ADEs increased surface DM1O_3_ across China by up to 4μgm^−3^. The summertime increase in DM1O_3_ results primarily from ADE-induced effects on atmospheric dynamics. It can thus be postulated that reducing ADEs will have the potential risk of increasing O_3_ in winter but will benefit the reduction in maximum O_3_ in summer.

Results from IPR analysis suggest that the ADE impacts exhibit strong vertical and diurnal variations. The ADE- induced decrease in modeled DM1O_3_ in January primarily results from ∆Photolysis, which reduced the chemical production of O_3_ in the near-ground layers. The increase in DM1O_3_ in July due to ADEs results from a weaker dry deposition sink as well as a stronger chemical source due to higher precursor concentrations in a more stable and shallow PBL. These impacts become stronger under higher PM_2.5_ concentrations when ADEs are larger.

The combined ADE impacts reduce O*_x_* in January due to ∆Photolysis but slightly increase O_*x*_ in July due to ∆Dynamics. OH is reduced by ADEs in January. However, midday OH concentrations during summertime show enhancements associated with both ∆Photolysis and ∆Dynamics, indicating a stronger midday atmospheric oxidizing capacity in July. An increased OH chain length in the near-ground layers is modeled both in January and July, indicating a stronger propagation efficiency of the chain reaction. In both January and July, *P*_HNO_3__ is increased and *P*_H_2_O_2__ is decreased due to ∆Dynamics, and both are reduced due to ∆Photolysis. The ratio of *P*_H_2_O_2__/*P*_HNO_3__ is decreased due to the combined impacts of ∆Dynamics and ∆Photolysis, indicating a shift towards more VOC-limited conditions due to ADEs in the near-ground layers during both January and July.

Thus aerosol direct effects on both photolysis rates as well as atmospheric dynamics can impact O_3_ formation rates and its local and regional distributions. Comparisons of integrated process rates suggest that the decrease in DM1O_3_ in January results from a larger net chemical sink due to ∆Photolysis, while the increase in DM1O_3_ in July is mostly associated with the slower removal due to reduced deposition velocity as well as a stronger photochemistry due to ∆Dynamics. The IRR analyses confirm that the process contributions from chemistry to DM1O_3_ can be influenced by both ∆Dynamics and ∆Photolysis. Reduced ventilation associated with ∆Dynamics enhances the precursor levels, which increase the chemical production rate of O*_x_* and OH, resulting in greater O_3_ chemical formation at noon during both January and July. One the other hand, reduced photolysis rates in ∆Photolysis result in lower O_3_ in January. How-ever, in July lower photolysis rates result in the accumulation of precursors during the morning hours, which eventually lead to higher O_3_ production at noon.

The comparison of integrated reaction rates from the various simulations also suggest that the increased OH_CL and the shift towards more VOC-limited conditions are mostly associated with the higher NO_2_ levels due to ADEs. This further emphasizes the importance of NO*_x_* controls in air pollution mitigation. Traditionally, the co-benefits from NO*_x_* control for ozone and PM reduction are mostly because NO*_x_* is a common precursor for both O_3_ and PM_2.5_. This study suggests that effective controls on NOx will not only gain direct benefits for O_3_ reduction but can also indirectly reduce peak O_3_ through weakening the ADEs from the reduced PM_2.5_, highlighting co-benefits from NO_*x*_ controls for achieving both O_3_ and PM_2.5_ reductions.

Reducing aerosols will have substantial impacts on ozone. The quantification of the aerosol influence on ozone is important to understand co-benefits associated with reductions in both particulate matter and ozone. This study focused on the evaluation of ADE impacts, which were not well quantified previously. However, the heterogeneous reactions associated with aerosols, as well as the impacts of emission controls of gaseous precursors on both aerosols and ozone also need to be studied in order to fully understand the influence of reducing aerosols on ambient ozone.

## Supplementary Material

Supp

## Figures and Tables

**Table 1.** Description of sensitivity simulations in this study.

Short name	Simulation description	Aerosol impacts on photolysis calculations	Aerosol impacts on radiation calculations
SimBL	Baseline simulation	No	No
SimNF	No aerosol feedback simulation	Yes	No
SimSF	Aerosol feedback simulation	Yes	Yes

**Table 2.** Comparison of model performance in ozone prediction across three simulations (monthly average of daily 1 h maxima).

Low PM_2.5_ (< 60 μg m^−3^)							High PM_2.5_ (< 60 μgm^−3^)				
	Region	OBS (μgm^−3^)	Normalized mean bias			∆-ADE* (μgm^−3^)	OBS (μgm^−3^)	Normalized mean bias			∆-ADE (μgm^−3^)
			SimSF	SimNF	SimBL			SimSF	SimNF	SimBL	
January	JJJ	62.52	3%	4%	5%	−1.05	37.02	22 %	36%	53 %	−11.36
	YRD	63.89	38%	41 %	43 %	−2.76	66.74	54%	59 %	67%	−8.85
	PRD	97.25	25 %	26 %	29 %	−4.52	122.61	6%	5%	9%	−4.63
	HUZ	47.67	172%	173%	193%	−10.17	67.29	107%	125%	142 %	−23.9
	SCH	88.63	−43%	−40%	−38%	−3.85	111.19	−5%	2%	8%	−13.78
	China	76.61	30%	31 %	34%	−2.96	62.68	42%	48 %	56%	−8.61

July	JJJ	159.27	−29%	−28%	−28%	−0.51	178.54	−25%	−25%	−25%	1.02
	YRD	171.04	−31 %	−31 %	−32%	0.84	233.13	−24%	−25%	−23%	−0.51
	PRD	129.02	−20%	−19%	−20%	−0.09	312.21	−44%	−45%	−46%	4.92
	HUZ	187.44	−36%	−37%	−37%	1.39	208.99	−27%	−28%	−29%	4.19
	SCH	163.81	−38%	−38%	−39%	0.77	191.19	−30%	−31 %	−31 %	1.18
	China	145.24	−28%	−28%	−28%	0.3	181.65	−25%	−25%	−25%	0.9

* ∆-ADE represents the O_3_ response to ADEs, which is calculated from the difference between SimSF and SimBL.
