# Supplementary material for: Impacts of aerosol direct effects on tropospheric ozone through changes in atmospheric dynamics and photolysis rates
Source: Atmos Chem Phys. Author manuscript; Available in PMC 2018 Aug 22. (PMC6104653; doi:10.5194/acp-17-9869-2017)
Supplement: Supp [file NIHMS982664-supplement-Supp.pdf]

Supplement of Atmos. Chem. Phys., 17, 9869–9883, 2017
https://doi.org/10.5194/acp-17-9869-2017-supplement
© Author(s) 2017. This work is distributed under
the Creative Commons Attribution 3.0 License.

Atmospheric Chemistry and Physics
Open Access

Supplement of

Impacts of aerosol direct effects on tropospheric ozone through changes in atmospheric dynamics and photolysis rates

Jia Xing et al.

Correspondence to: Shuxiao Wang (shxwang@tsinghua.edu.cn)

The copyright of individual parts of the supplement might differ from the CC BY 3.0 License.

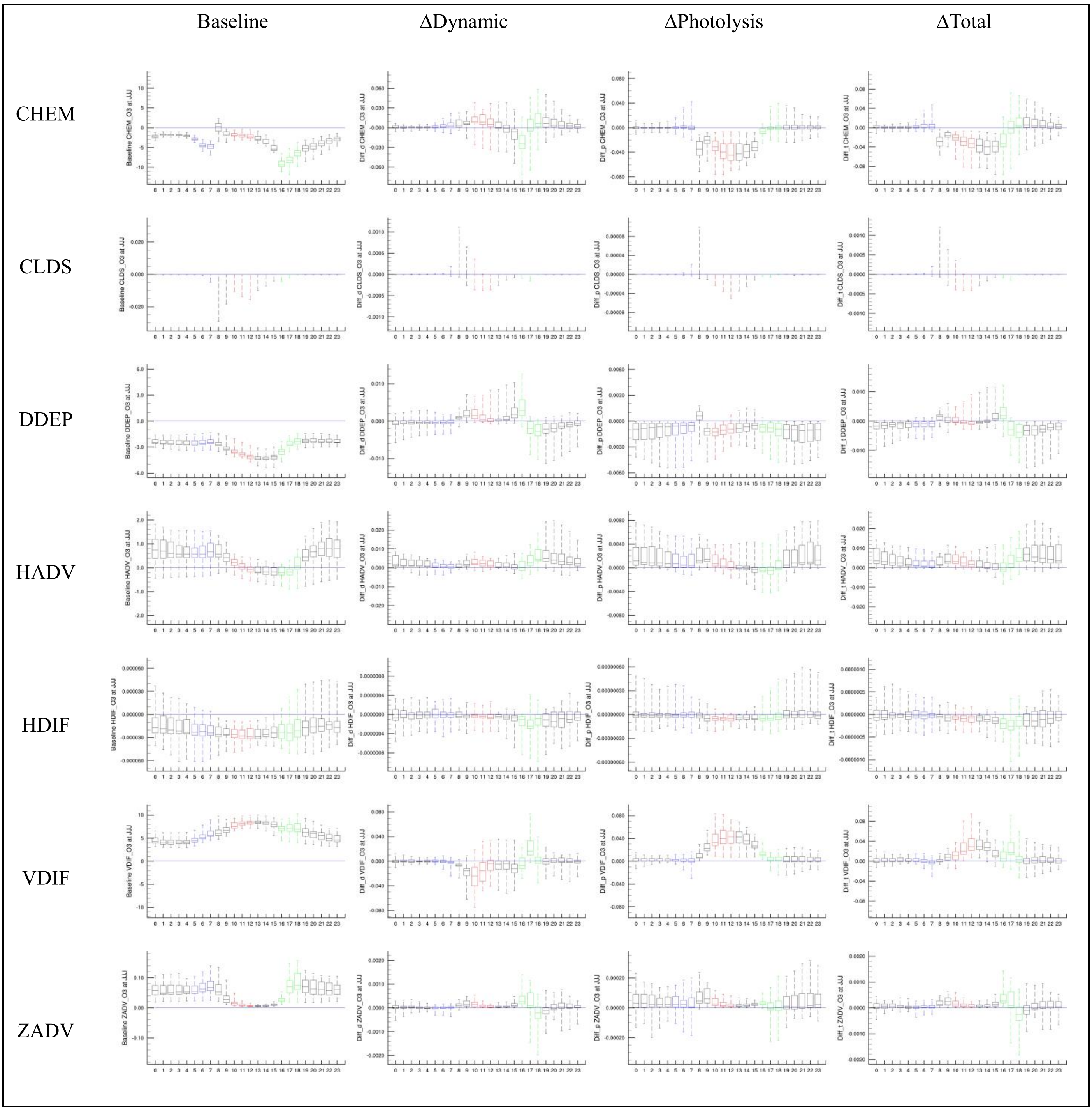
Baseline
ΔDynamic
ΔPhotolysis
ΔTotal
CHEM
CLDS
DDEP
HADV
HDIF
VDIF
ZADV
(a) January

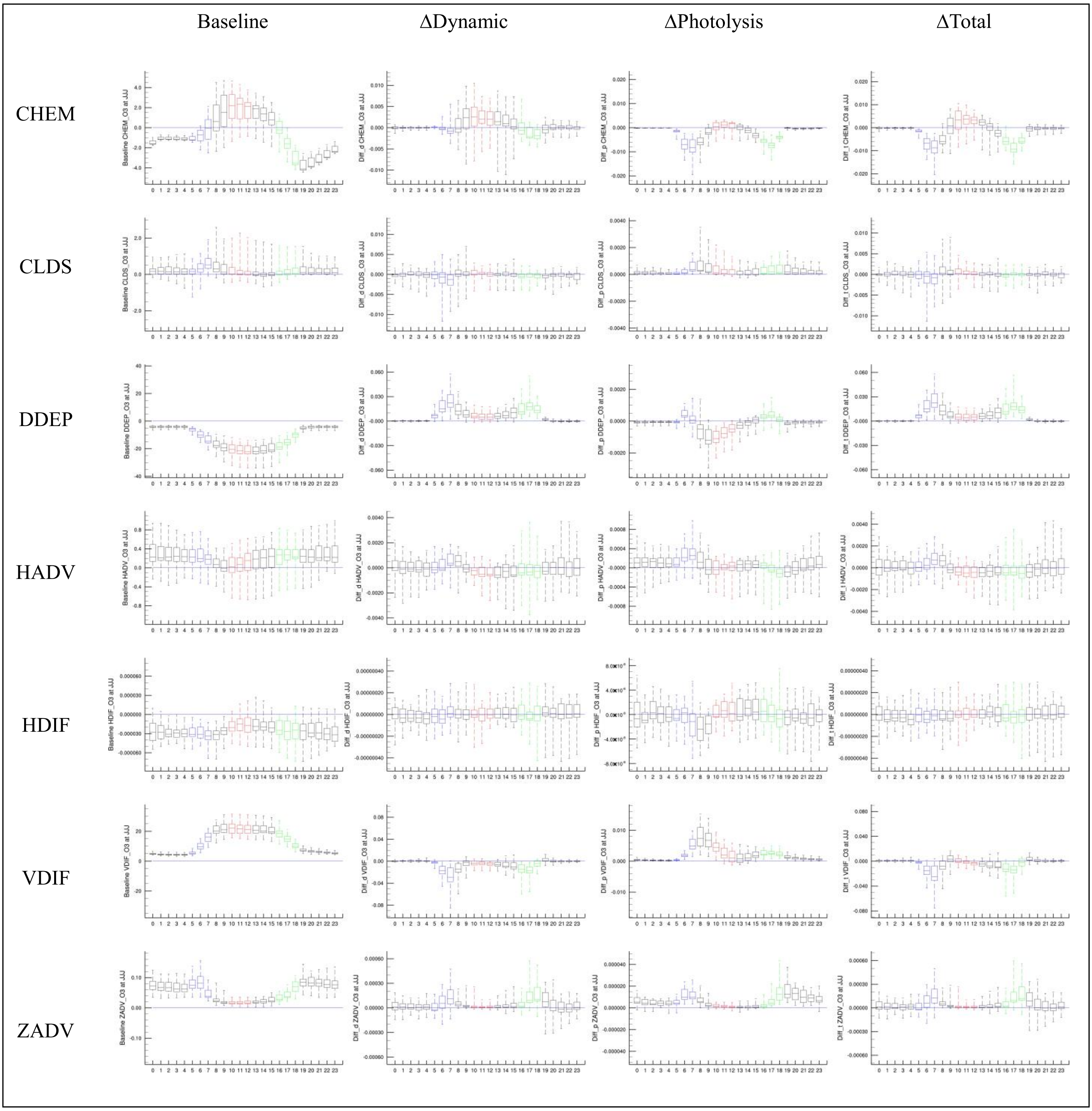

(b) July

Figure S1 Diurnal variation of integrated process contributions to surface O_3 concentration in JJJ (a: January; b: July; The calculation is based on the average of grid cells in JJJ; a. Baseline is the simulated O_3 in SimBL, unit: ppb hr^{-1}; b. ΔDynamic is the difference in normalized IPRs between SimSF and SimNF, unit: hr^{-1}; d. ΔPhotolysis is the difference in normalized IPRs between SimNF and SimBL, unit: hr^{-1}; c. ΔTotal is the difference in normalized IPRs between SimSF and SimBL, unit: hr^{-1}, colored bars represent three periods of early morning (blue), noon (red), and late afternoon (green))

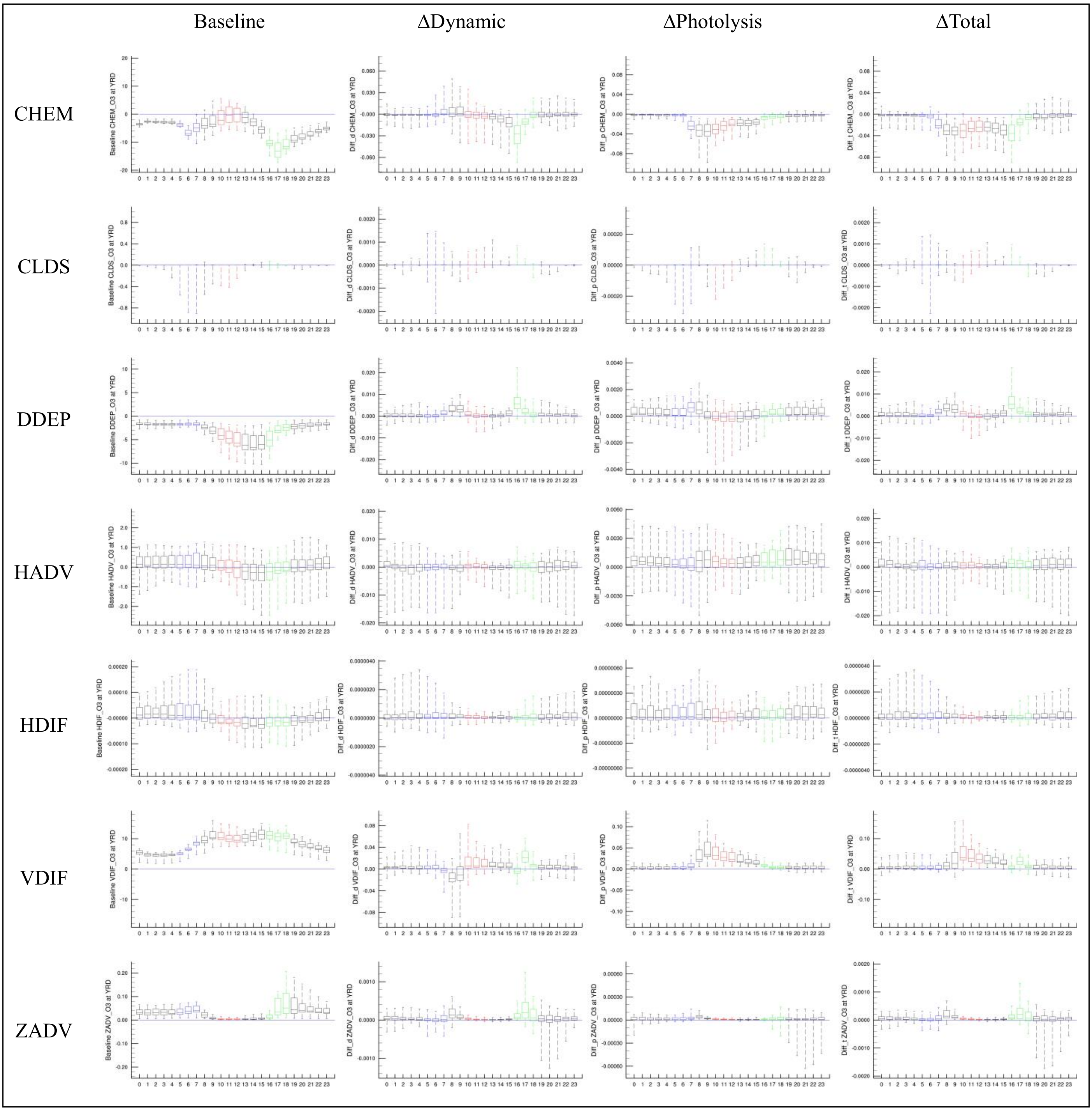

Baseline
ΔDynamic
ΔPhotolysis
ΔTotal
CHEM
CLDS
DDEP
HADV
HDIF
VDIF
ZADV

(a) January

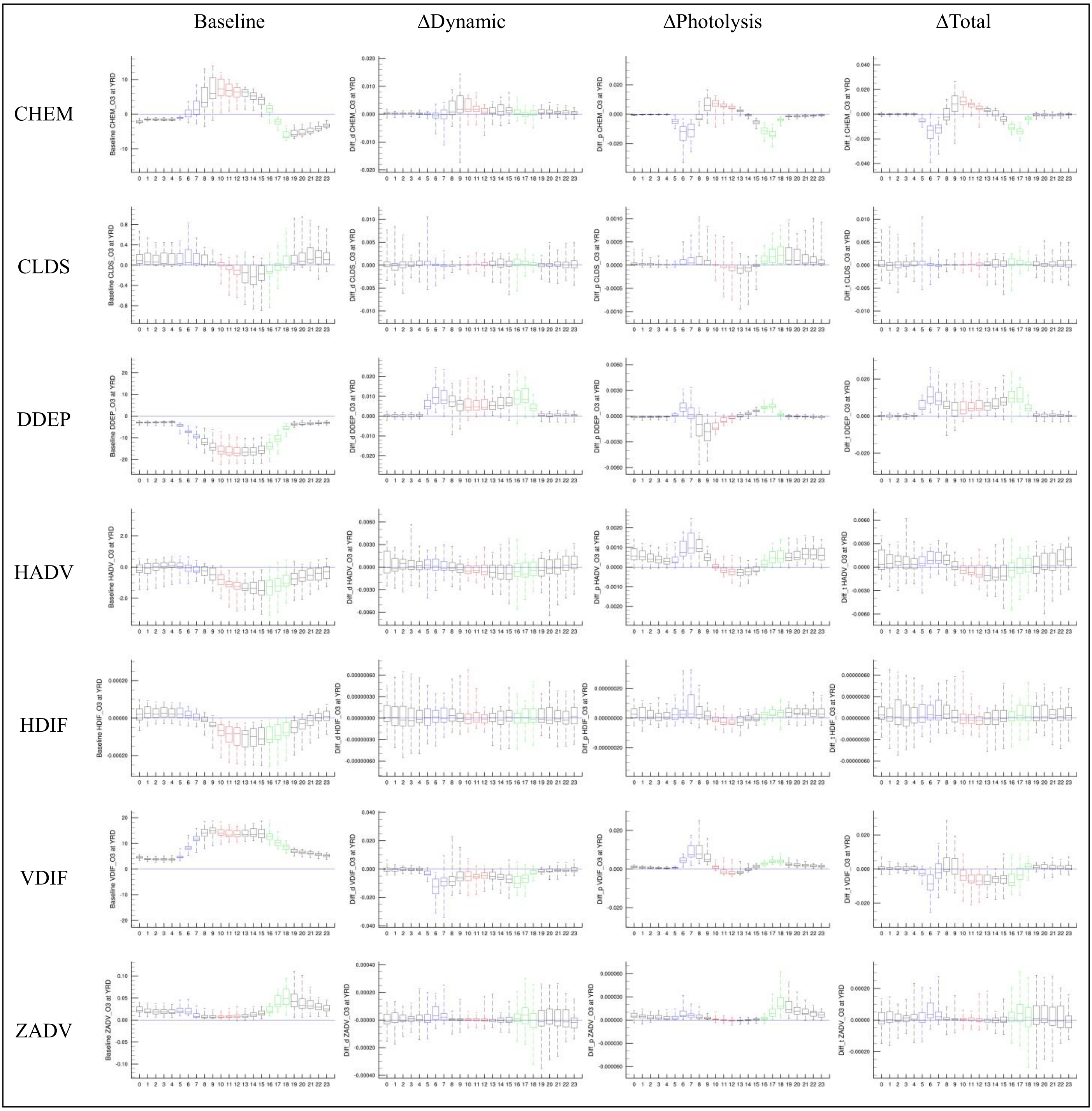

(b) July

Figure S2 same as Figure S1, but in YRD

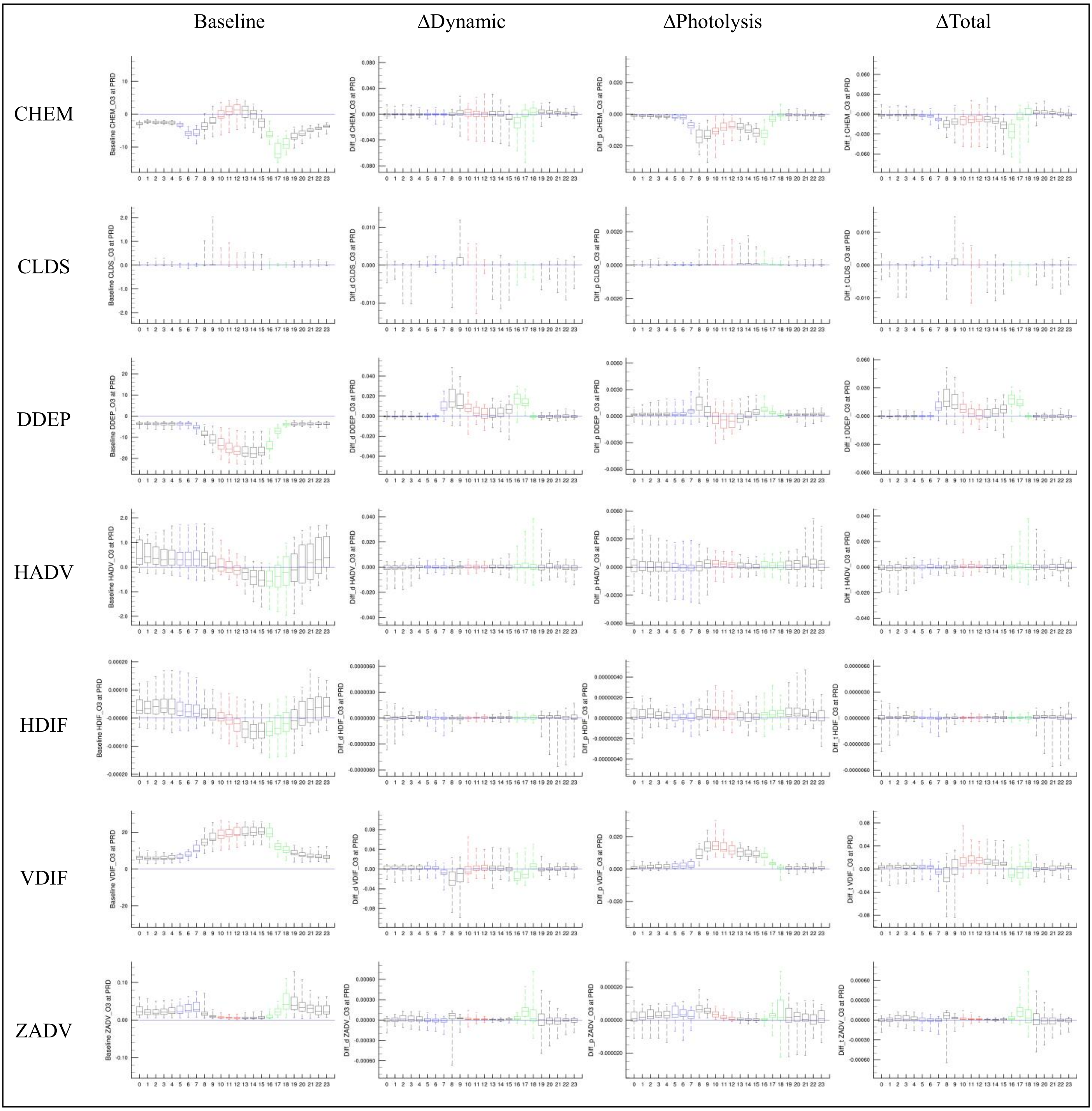
Baseline
ΔDynamic
ΔPhotolysis
ΔTotal
CHEM
CLDS
DDEP
HADV
HDIF
VDIF
ZADV

(a) January

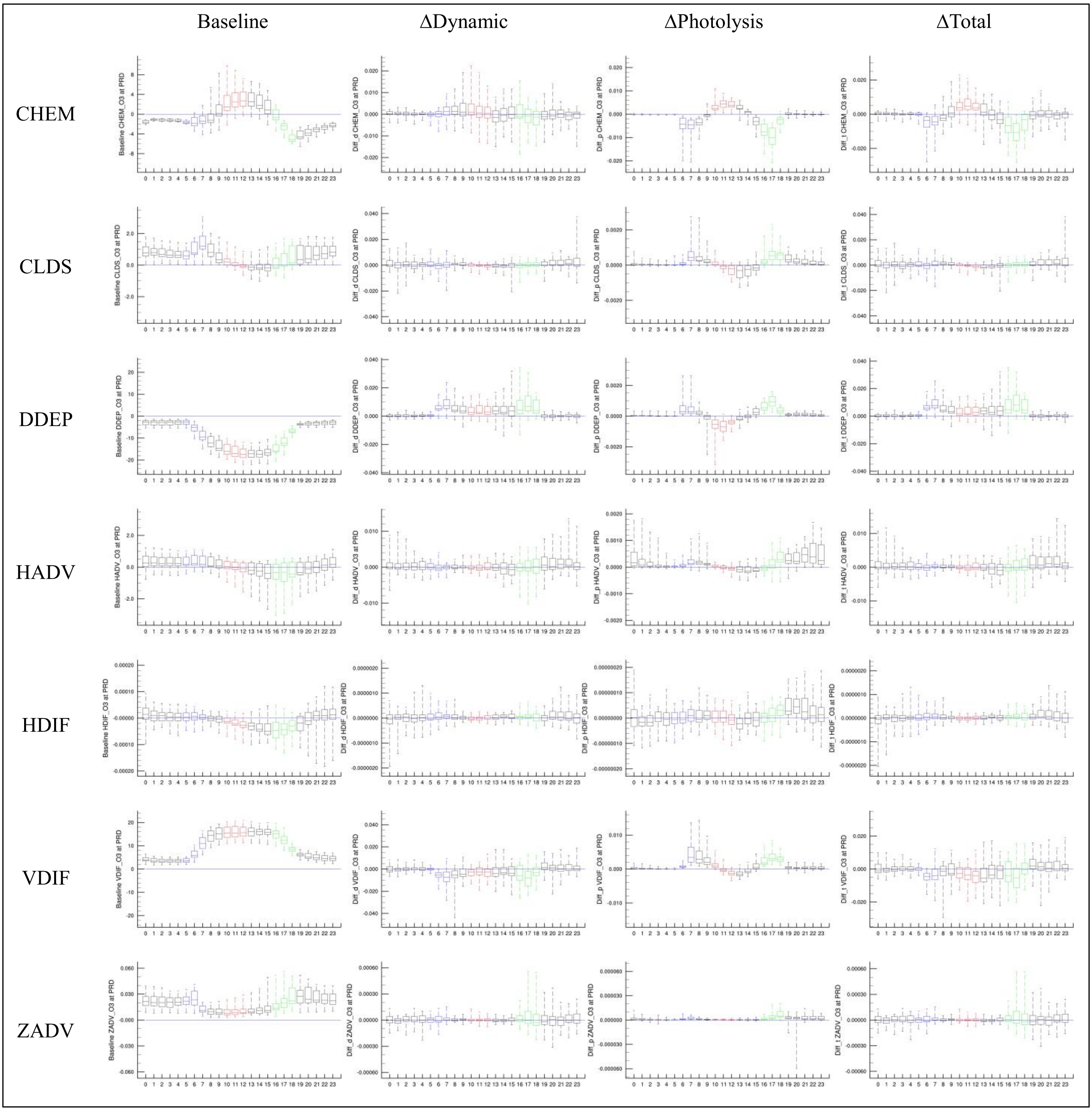

(b) July

Figure S3 same as Figure S1, but in PRD

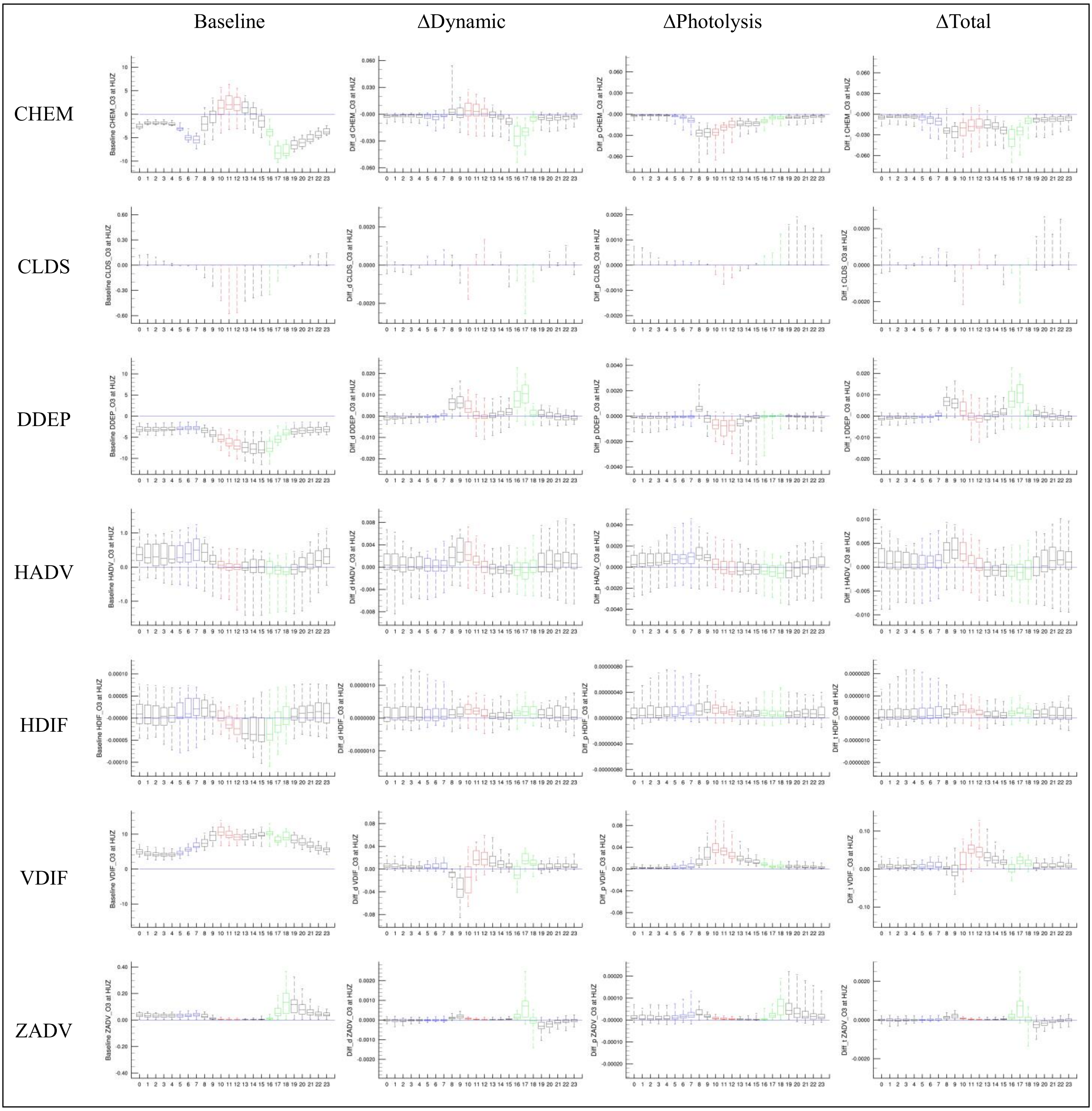
Baseline
ΔDynamic
ΔPhotolysis
ΔTotal
CHEM
CLDS
DDEP
HADV
HDIF
VDIF
ZADV

(a) January

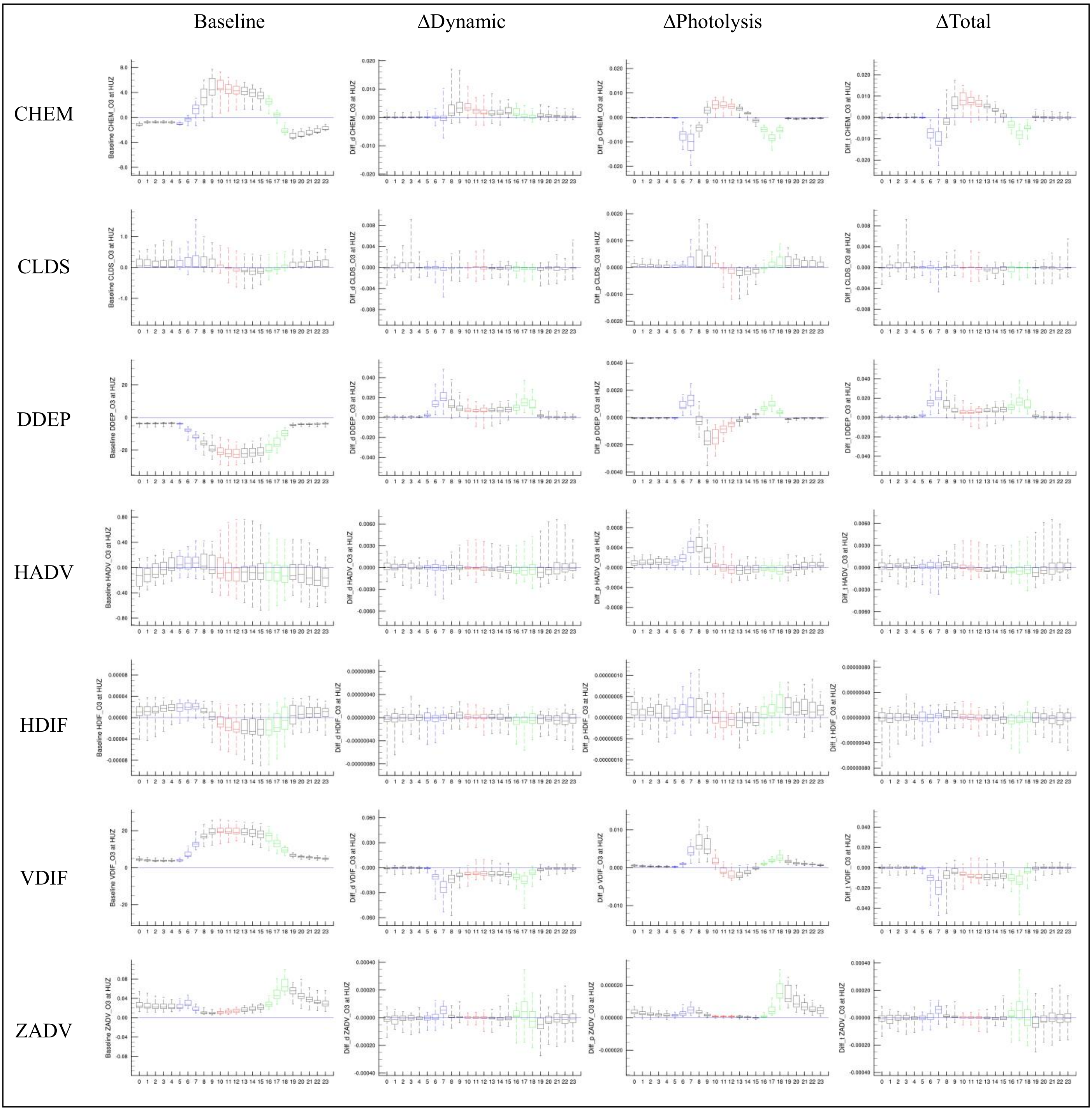

(b) July

Figure S4 same as Figure S1, but in HUZ

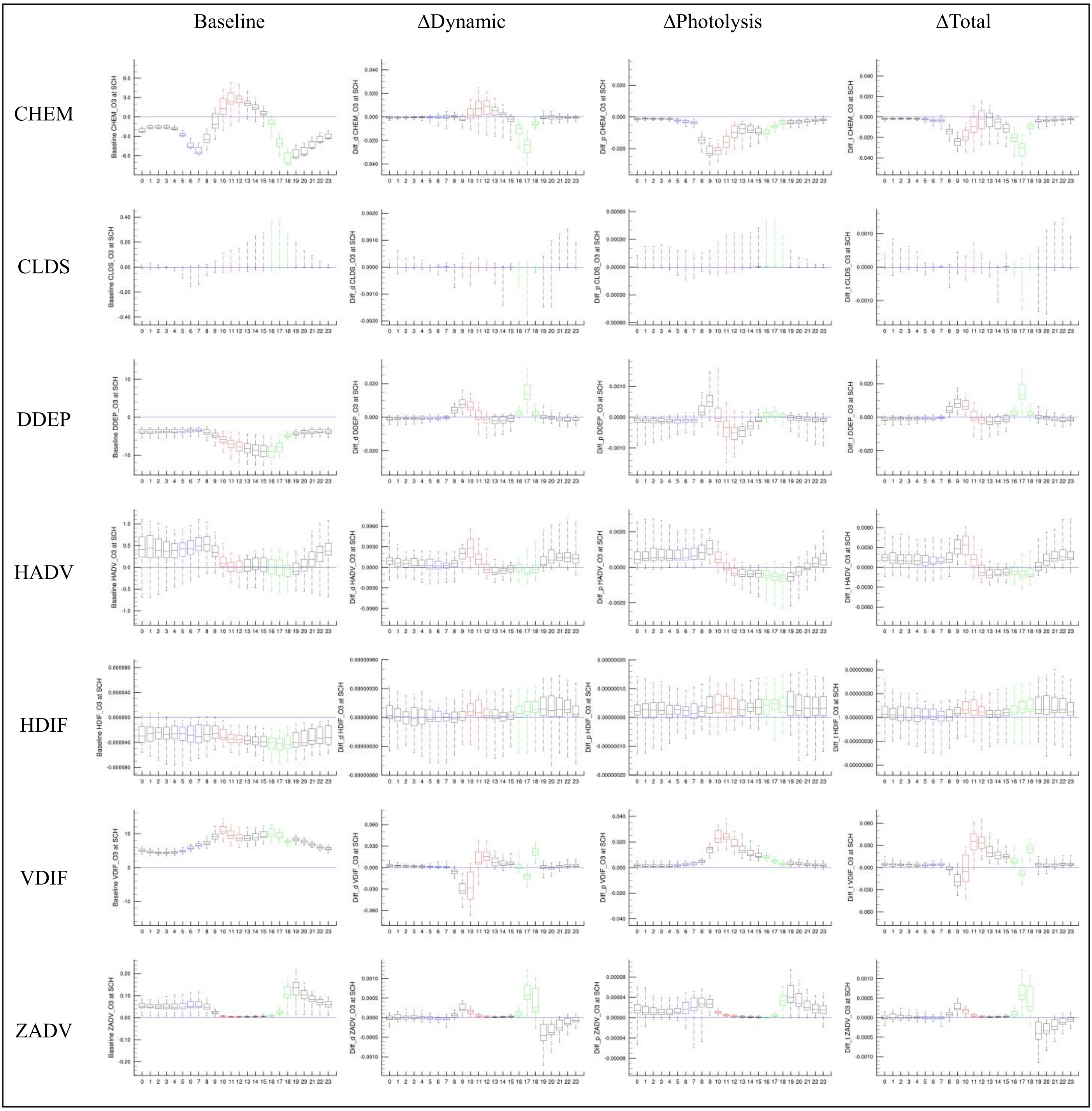
Baseline
ΔDynamic
ΔPhotolysis
ΔTotal
CHEM
CLDS
DDEP
HADV
HDIF
VDIF
ZADV

(a) January

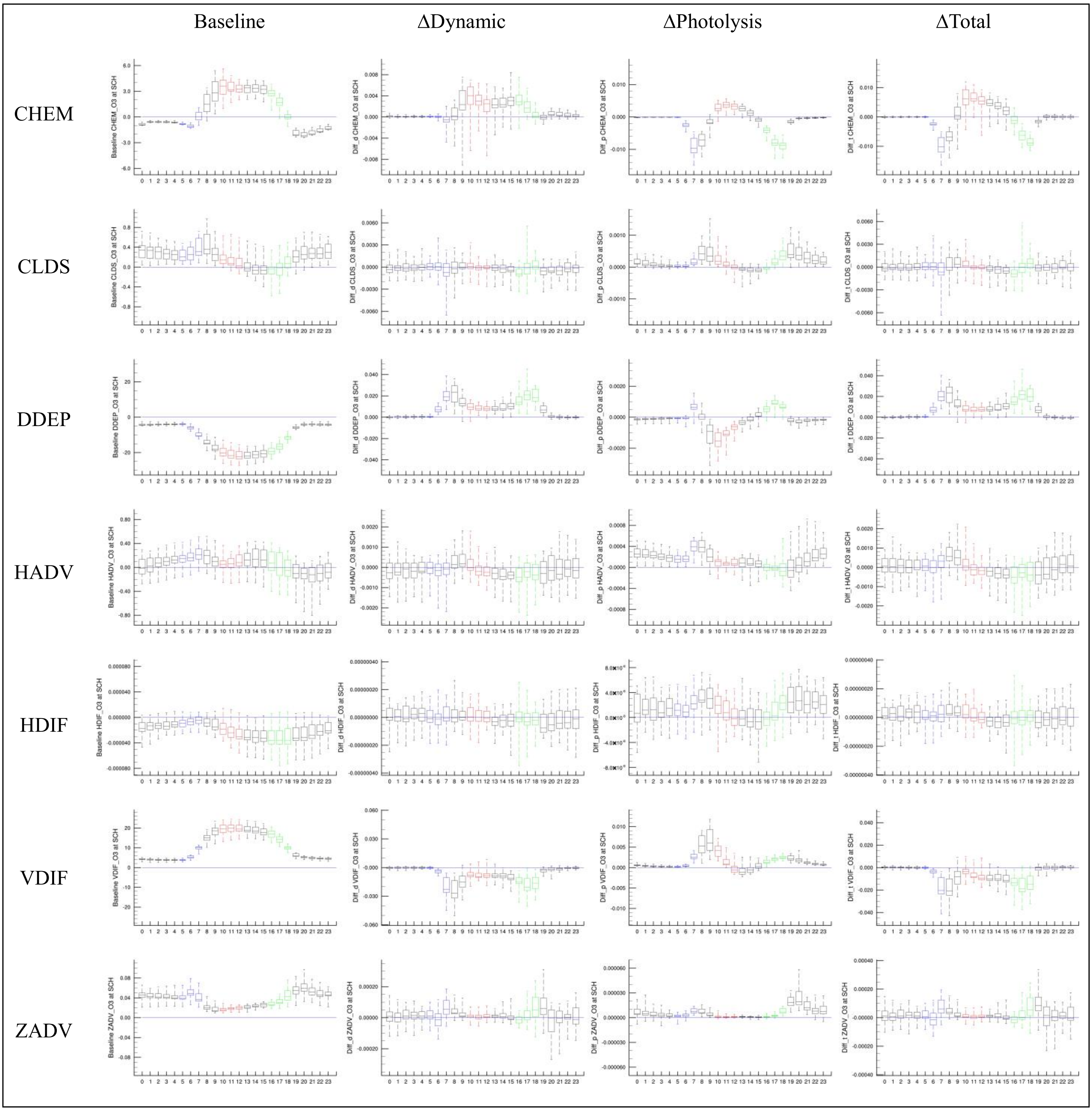

(b) July

Figure S5 same as Figure S1, but in SCH

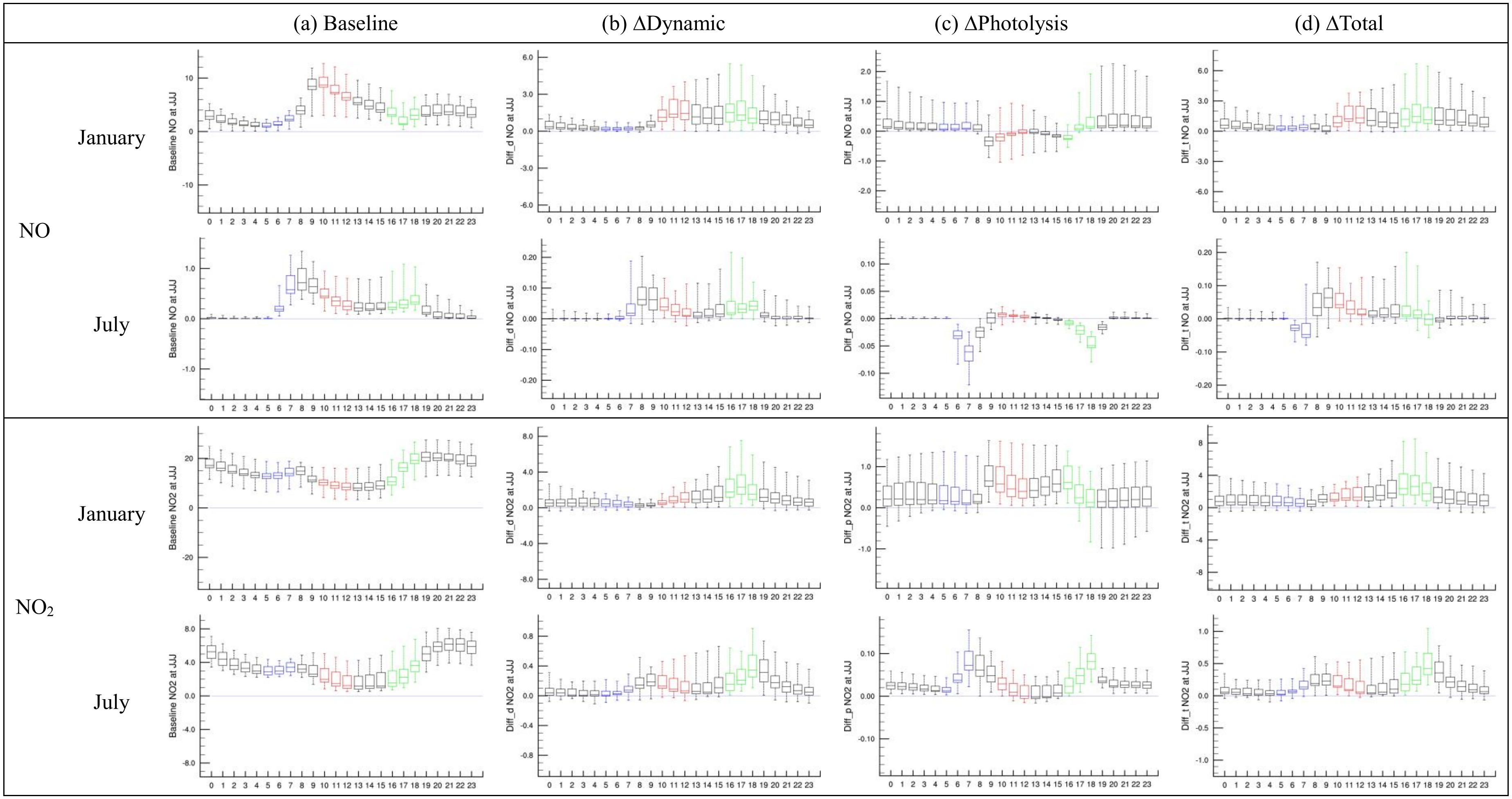

Figure S6 Diurnal variation of surface NO and NO_2 concentrations and their response to ADE in JJJ (Baseline is the simulated NO_X in SimBL, unit: ppb; b. ΔDynamic is the difference in the NO_X concentration between SimSF and SimNF, unit: ppb; c. ΔPhotolysis is the difference in the NO_X concentration between SimNF and SimBL, unit: ppb; d. ΔTotal is the difference in the NO_X concentration between SimSF and SimBL, unit: ppb)

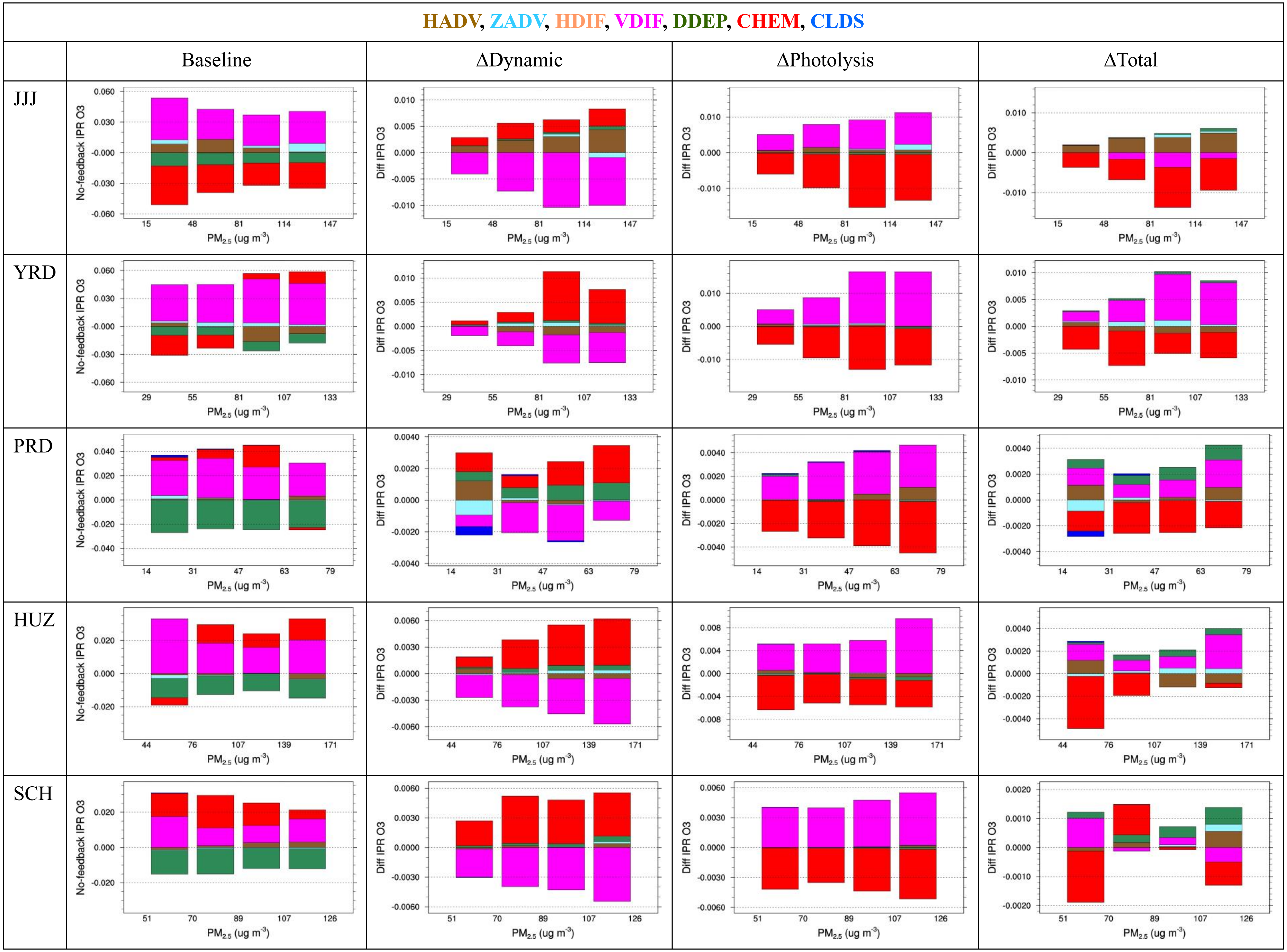
HADV, ZADV, HDIF, VDIF, DDEP, CHEM, CLDS
Baseline
ΔDynamic
ΔPhotolysis
ΔTotal
JJJ
YRD
PRD
HUZ
SCH
No-feedback IPR O3
Diff IPR O3
PM2.5 (ug m-3)

(a) January

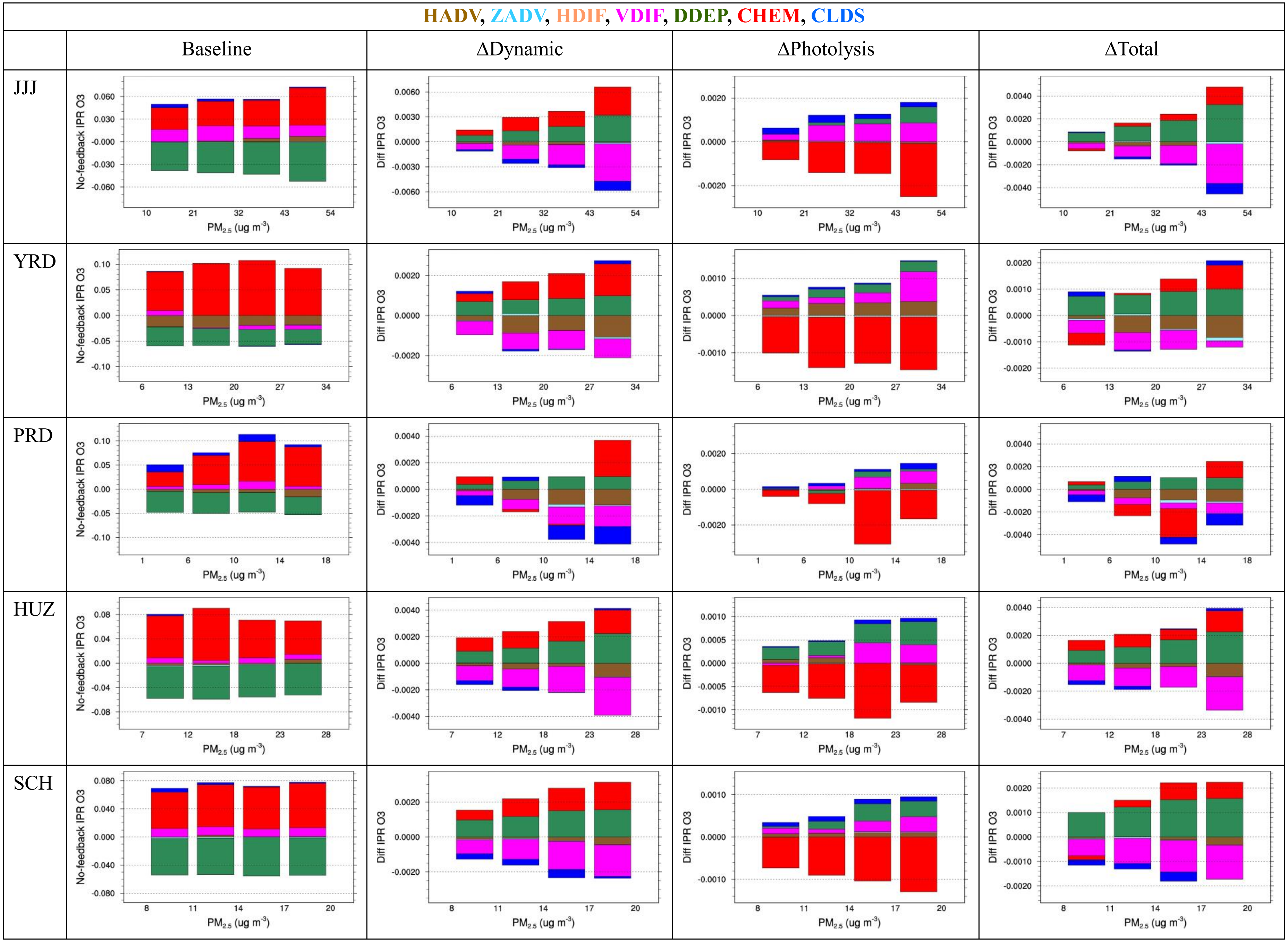

(b) July

Figure S7 Integrated process contributions to daytime near-ground-level O_3 under different $PM_{2.5}$ level in 5 regions (between the ground and 350m

AGL, model layer 1-5; a. Baseline is the normalized IPRs in SimBL, unit: hr^{-1}; b. ΔDynamic is the difference in normalized IPRs between SimSF and SimNF, unit: hr^{-1}; d. ΔPhotolysis is the difference in normalized IPRs between SimNF and SimBL, unit: hr^{-1}; c. ΔTotal is the difference in normalized IPRs between SimSF and SimBL, unit: hr^{-1})

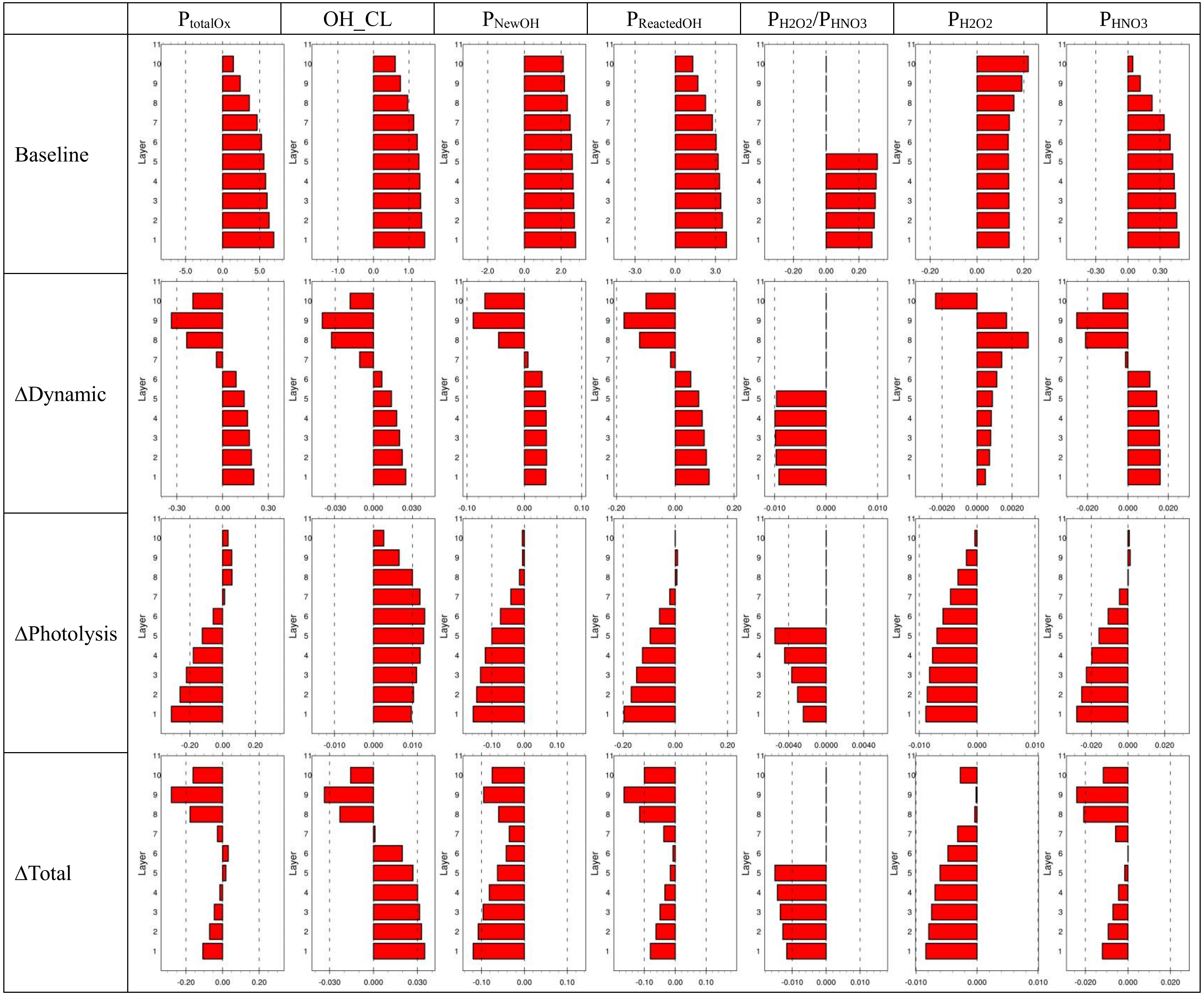
P totalOx
OH_CL
P NewOH
P ReactedOH
P H2O2/P HNO3
P H2O2
P HNO3
Baseline
ΔDynamic
ΔPhotolysis
ΔTotal
Layer

(a) January

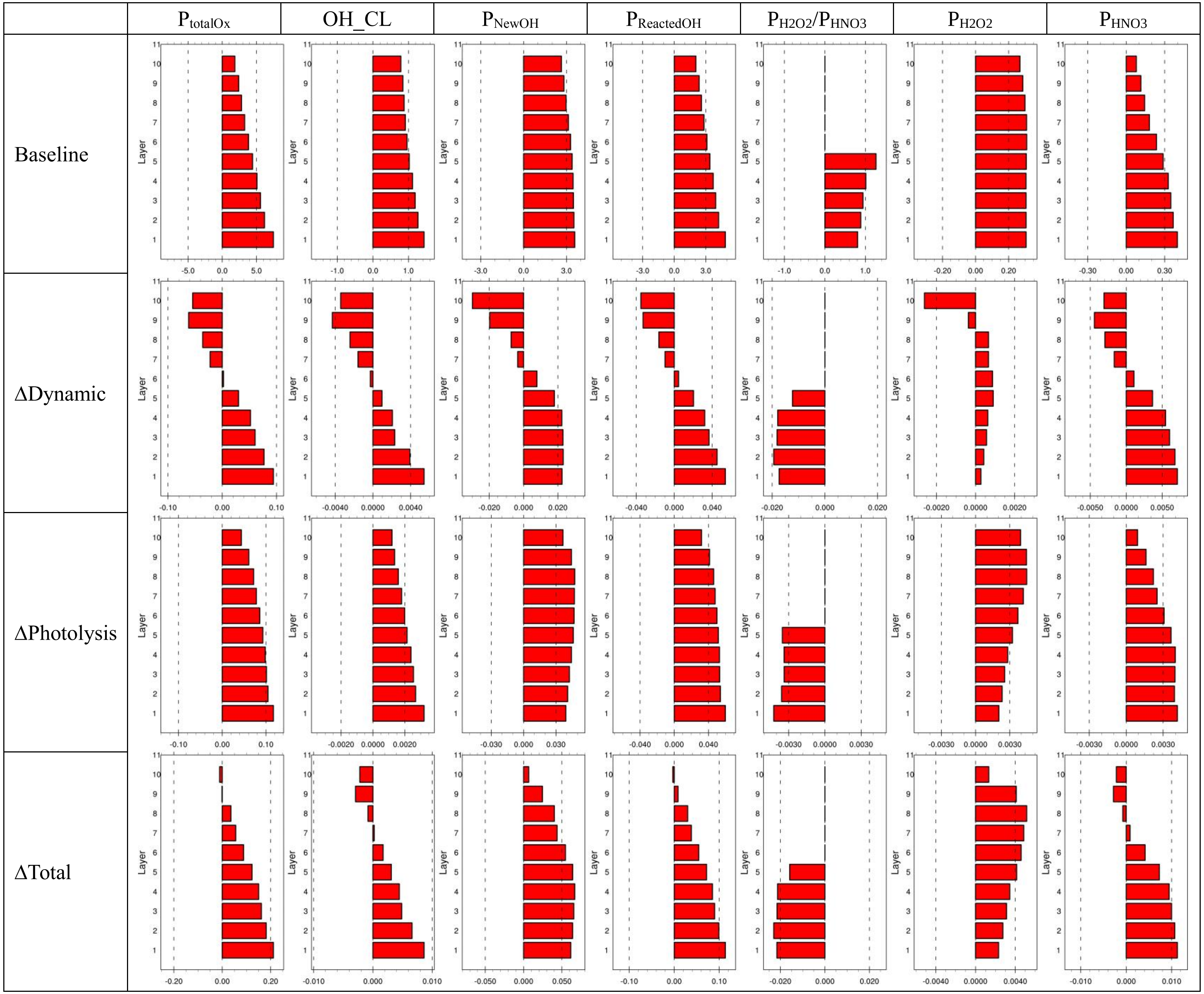

$P_{totalOx}$
OH_CL
P_{NewOH}
$P_{ReactedOH}$
P_{H2O2}/P_{HNO3}
P_{H2O2}
P_{HNO3}
Baseline
ΔDynamic
ΔPhotolysis
ΔTotal
Layer

(b) July

Figure S8 Vertical profile of integrated reaction rates in YRD at noon (a: January; b: July; full-layer heights above ground are 40, 96, 160, 241, 355, 503, 688, 884, 1100, 1357m; Baseline is the simulation in SimBL; ΔDynamic is the difference between SimSF and SimNF; ΔPhotolysis is the difference between SimNF and SimBL; ΔTotal is the difference between SimSF and SimBL; $P_{totalOx}$ is total O_x production rate, unit: ppb hr^{-1}; OH CL is OH chain length; P_{NewOH} is the production rate of new OH, unit: ppb hr^{-1}; $P_{ReactedOH}$ is the production rate of reacted OH, unit: ppb hr^{-1}; P_{H2O2} is the production rate of H_2O_2, unit: ppb hr^{-1}; P_{HNO3} is the production rate of HNO_3, unit: ppb hr^{-1}; the ratio of P_{H2O2}/P_{HNO3} is only shown for layer 1-5)

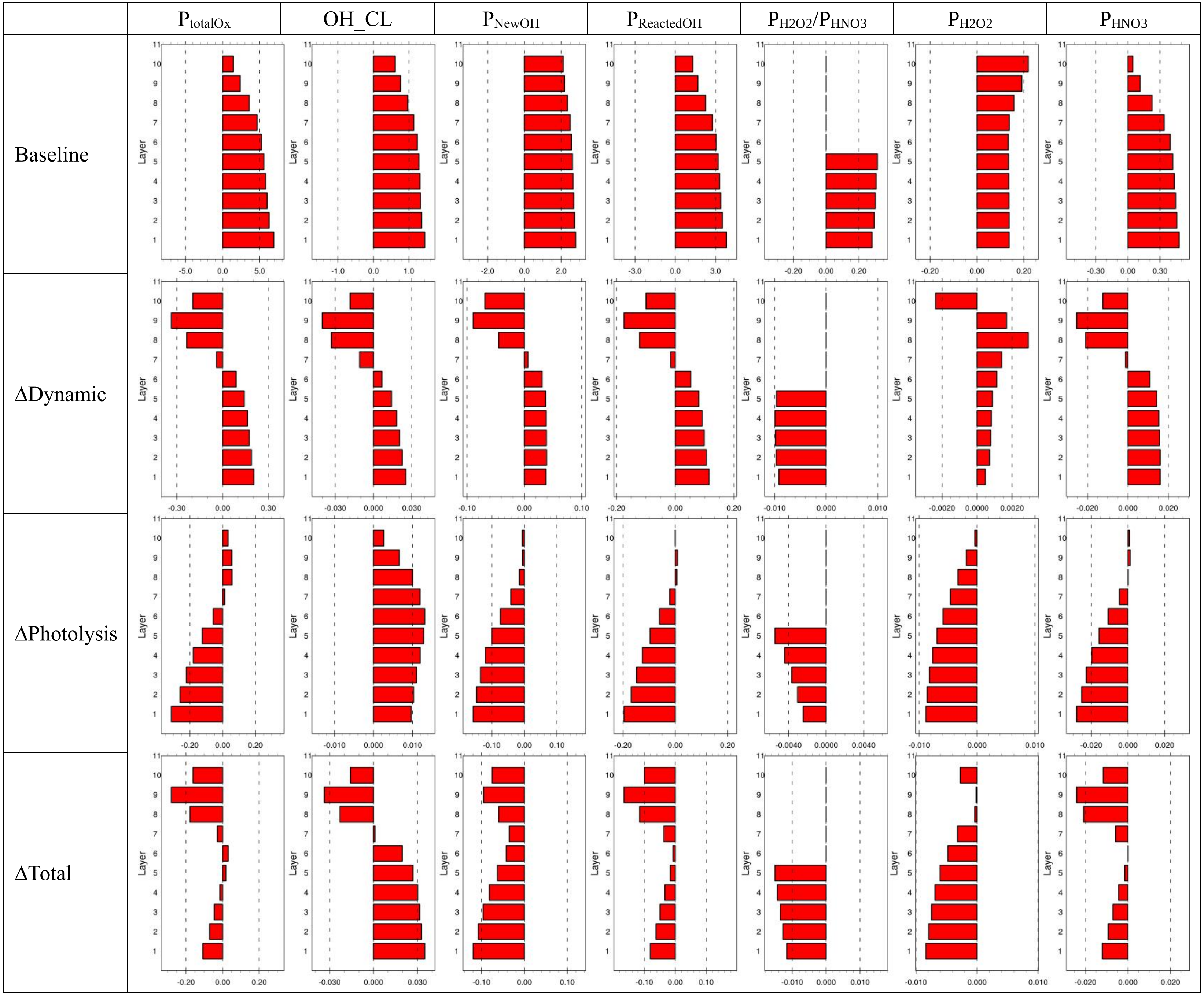

P_totalOx
OH_CL
P_NewOH
P_ReactedOH
P_H2O2/P_HNO3
P_H2O2
P_HNO3
Baseline
ΔDynamic
ΔPhotolysis
ΔTotal
Layer

(a) January

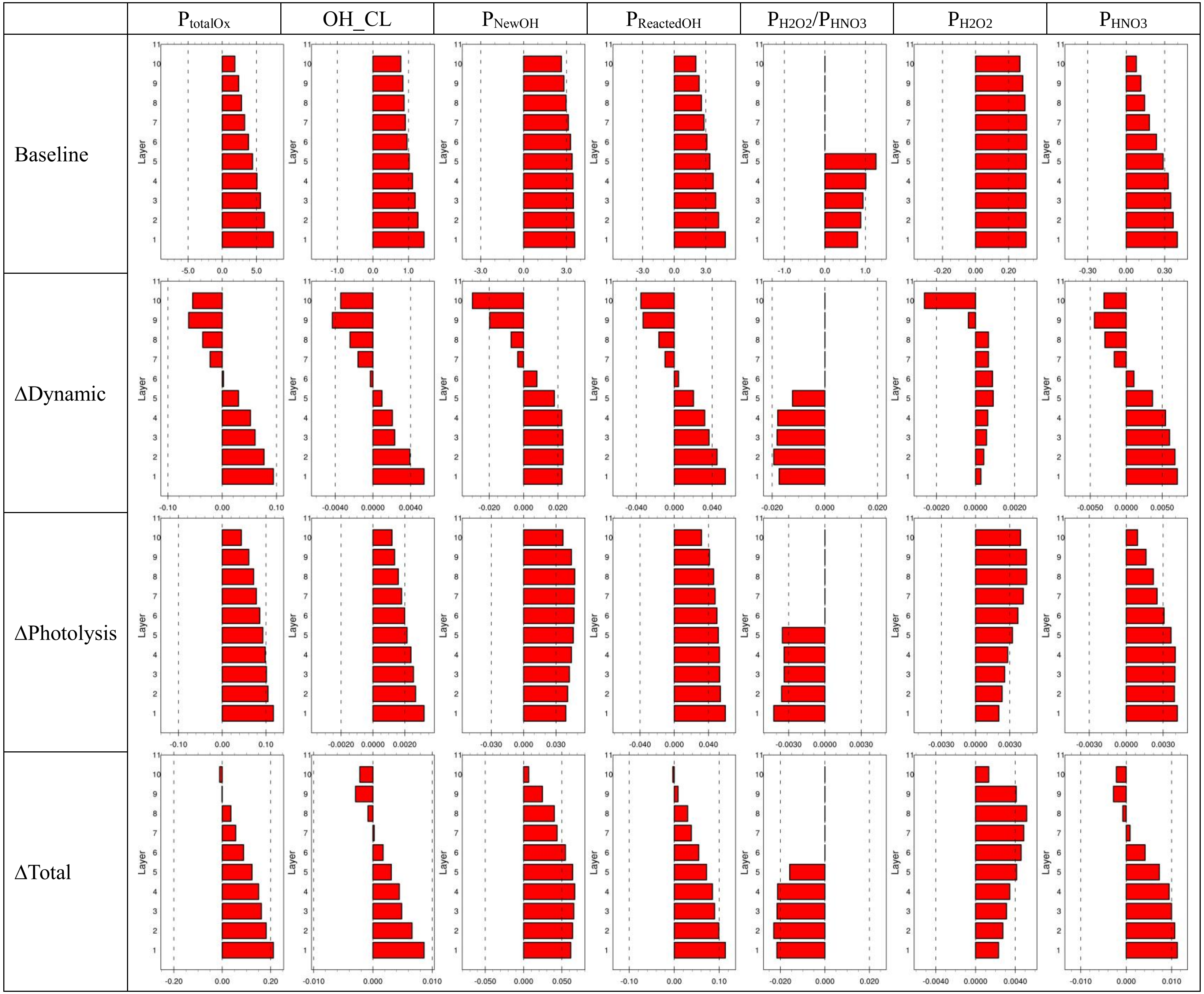

$P_{totalOx}$
OH_CL
P_{NewOH}
$P_{ReactedOH}$
P_{H2O2}/P_{HNO3}
P_{H2O2}
P_{HNO3}
Baseline
ΔDynamic
ΔPhotolysis
ΔTotal
Layer

(b) July

Figure S9 Same as Figure S8, but in PRD

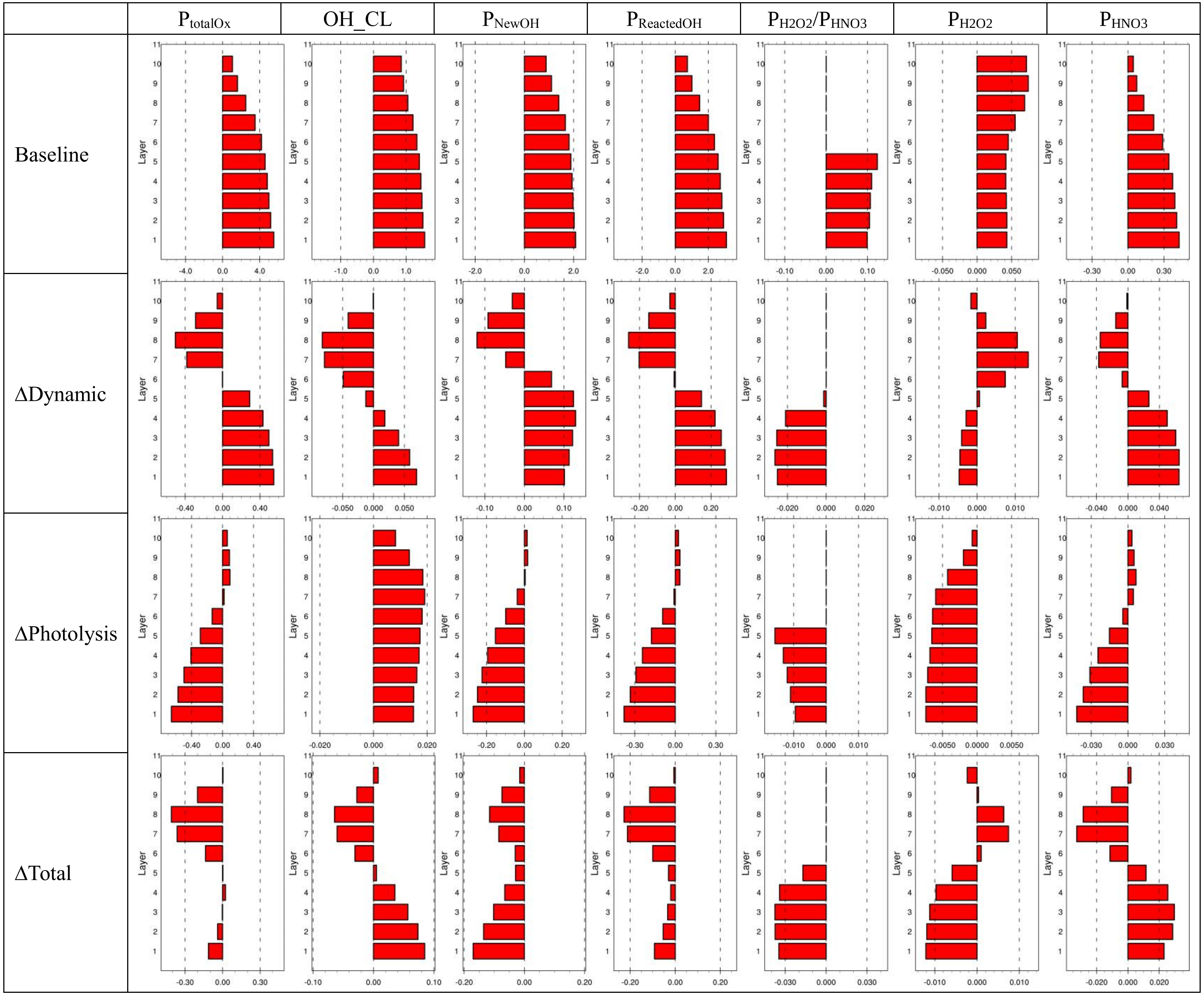
$P_{totalOx}$
OH_CL
P_{NewOH}
$P_{ReactedOH}$
P_{H2O2}/P_{HNO3}
P_{H2O2}
P_{HNO3}
Baseline
ΔDynamic
ΔPhotolysis
ΔTotal
Layer

(a) January

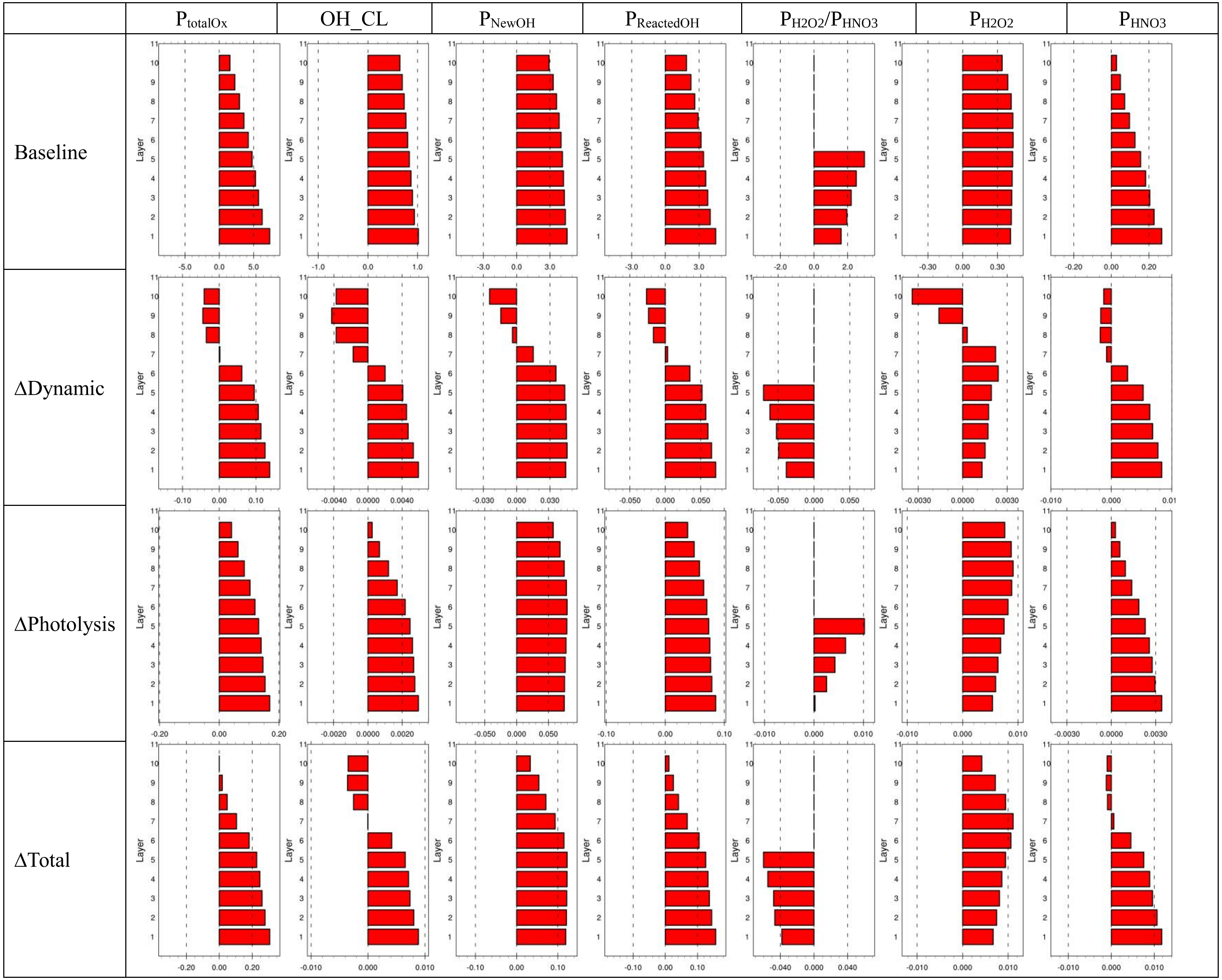

P totalOx
OH_CL
P NewOH
P ReactedOH
P H2O2/P HNO3
P H2O2
P HNO3
Baseline
ΔDynamic
ΔPhotolysis
ΔTotal
Layer

(b) July

Figure S10 Same as Figure S8, but in HUZ

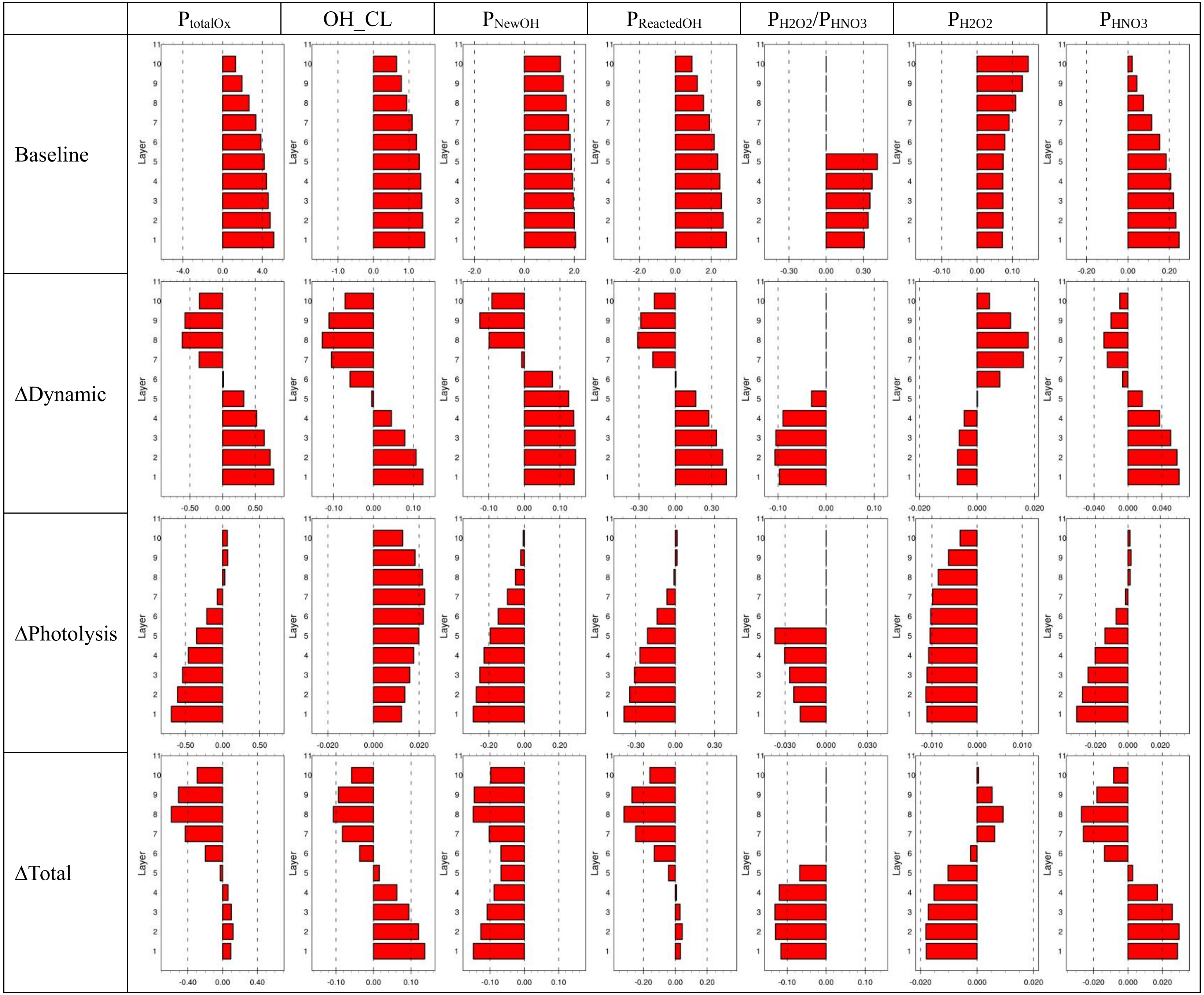
$P_{totalOx}$
OH_CL
P_{NewOH}
$P_{ReactedOH}$
P_{H2O2}/P_{HNO3}
P_{H2O2}
P_{HNO3}
Baseline
ΔDynamic
ΔPhotolysis
ΔTotal
Layer

(a) January

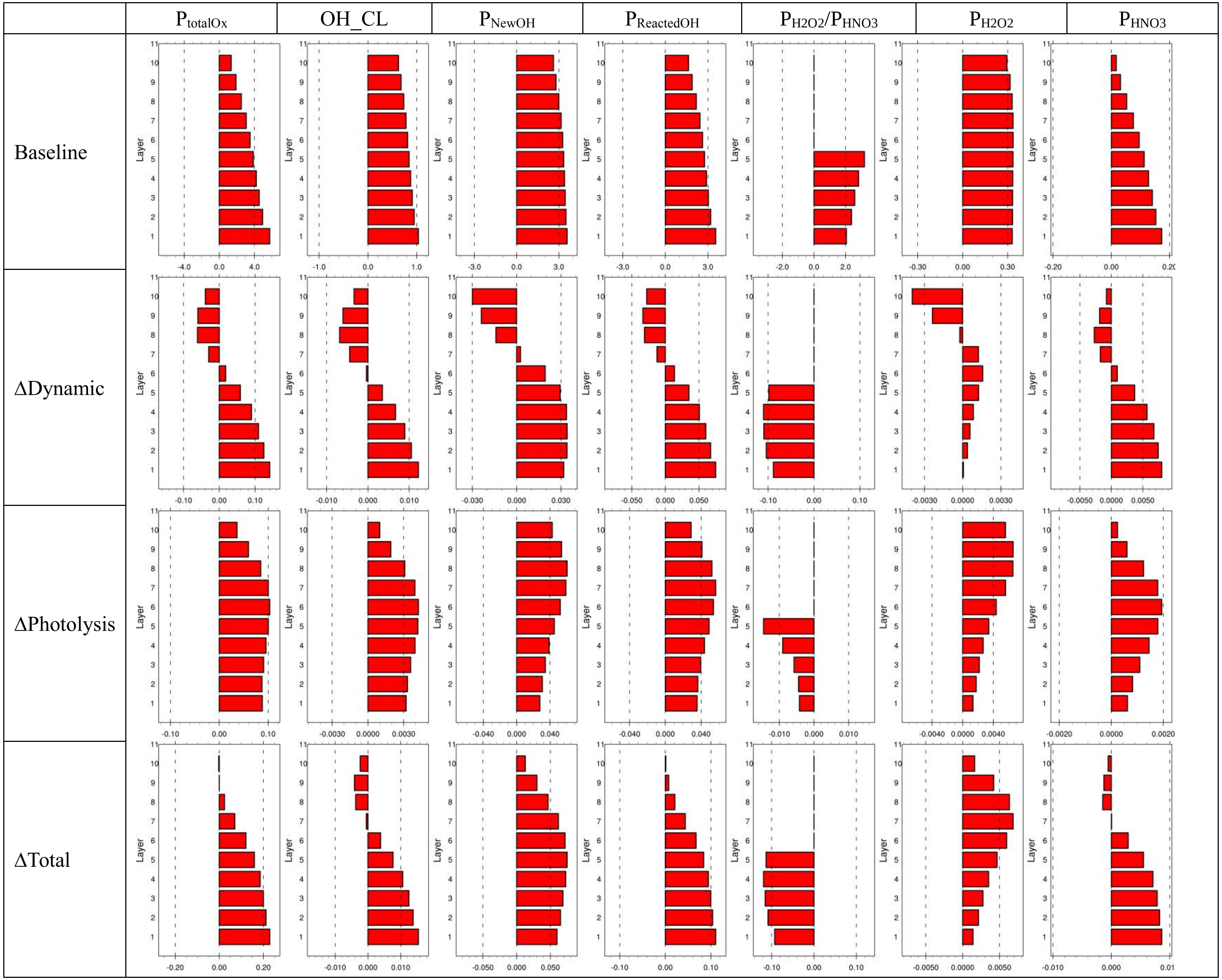
P totalOx
OH_CL
P NewOH
P ReactedOH
P H2O2/P HNO3
P H2O2
P HNO3
Baseline
ΔDynamic
ΔPhotolysis
ΔTotal
Layer

(b) July

Figure S11 Same as Figure S8, but in SCH
